# Genetic analysis of wheat sensitivity to the ToxB fungal effector from *Pyrenophora tritici-repentis*, the causal agent of tan spot

**DOI:** 10.1007/s00122-019-03517-8

**Published:** 2020-01-08

**Authors:** Beatrice Corsi, Lawrence Percival-Alwyn, Rowena C. Downie, Luca Venturini, Elyce M. Iagallo, Camila Campos Mantello, Charlie McCormick-Barnes, Pao Theen See, Richard P. Oliver, Caroline S. Moffat, James Cockram

**Affiliations:** 1 John Bingham Laboratory, NIAB, Huntingdon Road, Cambridge, CB3 0LE UK; 2 Plant Sciences Department, University of Cambridge, Cambridge, UK; 3 Life Sciences Department, Natural History Museum, Cromwell Road, London, SW7 5BD UK; 4 Centre for Crop and Disease Management, School of Molecular and Life Sciences, Curtin University, Perth, Australia; 5 Faculty of Biology, Medicine and Health, The University of Manchester, Oxford Road, Manchester, M13 9PL UK; 6 Present Address: Genetracer Biotech, Calle Albert Einstein 22, 39011 Santander, Spain

## Abstract

**Key message:**

Genetic mapping of sensitivity to the *Pyrenophora tritici-repentis* effector ToxB allowed development of a diagnostic genetic marker, and investigation of wheat pedigrees allowed transmission of sensitive alleles to be tracked.

**Abstract:**

Tan spot, caused by the necrotrophic fungal pathogen *Pyrenophora tritici-repentis*, is a major disease of wheat (*Triticum aestivum*). Secretion of the *P. tritici-repentis* effector ToxB is thought to play a part in mediating infection, causing chlorosis of plant tissue. Here, genetic analysis using an association mapping panel (*n* = 480) and a multiparent advanced generation intercross (MAGIC) population (*n* founders = 8, *n* progeny = 643) genotyped with a 90,000 feature single nucleotide polymorphism (SNP) array found ToxB sensitivity to be highly heritable (*h*^2^ ≥ 0.9), controlled predominantly by the *Tsc2* locus on chromosome 2B. Genetic mapping of *Tsc2* delineated a 1921-kb interval containing 104 genes in the reference genome of ToxB-insensitive variety ‘Chinese Spring’. This allowed development of a co-dominant genetic marker for *Tsc2* allelic state, diagnostic for ToxB sensitivity in the association mapping panel. Phenotypic and genotypic analysis in a panel of wheat varieties post-dated the association mapping panel further supported the diagnostic nature of the marker. Combining ToxB phenotype and genotypic data with wheat pedigree datasets allowed historic sources of ToxB sensitivity to be tracked, finding the variety ‘Maris Dove’ to likely be the historic source of sensitive *Tsc2* alleles in the wheat germplasm surveyed. Exploration of the *Tsc2* region gene space in the ToxB-sensitive line ‘Synthetic W7984’ identified candidate genes for future investigation. Additionally, a minor ToxB sensitivity QTL was identified on chromosome 2A. The resources presented here will be of immediate use for marker-assisted selection for ToxB insensitivity and the development of germplasm with additional genetic recombination within the *Tsc2* region.

**Electronic supplementary material:**

The online version of this article (10.1007/s00122-019-03517-8) contains supplementary material, which is available to authorized users.

## Introduction

Tan spot, also known as yellow (leaf) spot, is a major fungal disease of wheat (*Triticum aestivum* L.). It is caused by the necrotrophic fungus *Pyrenophora tritici-repentis* (Died.) Drechs. (abbreviated here as *Ptr*; syn, *Dreschlera tritici-repentis* [*Dtr*]) and typified by necrotic lesions as well as regions of chlorosis on infected leaves, which result in reduced leaf photosynthetic area. This typically results in a 5–10% reduction in grain yield, although losses can reach 50% under favourable conditions (De Wolf et al. 1998). Tan spot is recognised as a major disease in a number of wheat-growing areas, including Europe, South America, Canada and Australia (Annone 1998; Ciuffetti et al. 2014; Savary et al. 2019). A complex *Ptr* race structure has been defined, with screening against a differential set of wheat varieties allowing at least eight races to be described, termed race 1–8 (Lamari et al. 2003). Necrotrophic effectors (previously termed ‘host-selective toxins’) mediate the interaction between a given *Ptr* race and its susceptible differential host line. In contrast to the classical gene-for-gene model, whereby interaction of avirulence effectors with host resistance gene complexes confers resistance, the tan spot host–pathogen system appears to be largely governed in an inverse gene-for-gene manner, whereby effector sensitivity is conferred by a single dominant host gene (Tan et al. 2010). The host genotype specificity of effectors makes them important factors in disease development, and the removal of host sensitivity genes is a priority for breeding efforts.

To date, three *Ptr* effectors have been described and demonstrated to be pathogenicity factors: ToxA, ToxB and ToxC. Each *Ptr* race is largely differentiated by its expression of one or a combination of these three effectors (Lamari et al. 2003). ToxA was the first *Ptr* effector to be isolated and is the most well studied (Ballance et al. 1989; Tomas et al. 1990; Faris et al. 1996). The majority of *Ptr* isolates worldwide produce ToxA (Friesen et al. 2005) comprising of races 1, 2, 7 and 8. ToxA triggers necrosis in wheat lines carrying susceptible alleles at the *Tsn1* locus. The mature PtrToxA protein encodes a 13.2 kDa peptide containing a fibronectin type III-like domain (Balance et al. 1996; Ciuffetti et al. 1997) and includes an arginyl-glycyl-aspartic acid (RGD) motif thought to be important for receptor binding and internalisation into wheat cells (Meinhardt et al. 2002; Manning et al. 2008). The *ToxA* gene is thought to have been transferred to *Ptr* via horizontal gene transfer from another wheat necrotrophic pathogen, *Parastagonospora nodorum* (Friesen et al. 2006). In wheat, the *Tsn1* locus confers sensitivity to ToxA from both *Ptr* and *P. nodorum*. *Tsn1* encodes a predicted protein containing a nucleotide-binding site leucine-rich repeat (NBS-LRR) domain and a serine/threonine protein kinase (S/TPK) domain (Faris et al. 2010). Wheat varieties insensitive to ToxA predominantly carry a complete deletion of *Tsn1*. However, while *Tsn1* is necessary to mediate ToxA recognition, several yeast‐2‐hybrid studies have reported different interacting proteins in the host. Early work showed that ToxA interacts with plastocyanin (Tai et al. 2007) and the chloroplast ToxABP1 protein (Manning et al. 2007), both of which may promote the induction of reactive oxygen species leading to cell death. More recently ToxA has been demonstrated to interact with the wheat pathogencitiy-related PR-1-5 protein in a highly specific manner (Lu et al. 2014). ToxA-induced necrosis was enhanced by co-infiltration of both ToxA and PR-1-5 into sensitive wheat lines, but not with a non-interacting PR-1-5^N141A^ mutant (Lu et al. 2014). Therefore, the interaction of ToxA with the PR-1-5 protein appears to play a role in promoting necrosis in *Tsn1*-containing wheat. The role of ToxA as a key tan spot disease determinant has been demonstrated in interactions with many but not all wheat cultivars (Ciuffetti et al. 1997). Recent work screening Australian wheat lines with a *Ptr* mutant carrying a deletion of *ToxA* has further highlighted the importance of ToxA in tan spot disease (See et al. 2018). Disease levels were significantly reduced on about one third of the *Tsn1* wheat lines. The observation that disease levels did not always decrease highlights that while ToxA is a key determinant in tan spot disease, it is not the whole story.

Compared to the ToxA-*Tsn1* interaction which results in rapid necrosis, the ToxB-*Tsc2* and ToxC-*Tsc1* interactions both result in slower chlorotic responses in sensitive wheat lines. ToxC is produced by *Ptr* races 1, 3, 6 and 8 (Strelkov and Lamari 2003). Partial culture filtrate purification based on gel filtration, ion exchange and reverse-phase chromatography indicated that ToxC is a polar, non-ionic, low molecular mass molecule (Effertz et al. 2002). ToxC-induced chlorosis was observed on wheat cultivars carrying sensitive alleles at the *Tsc1* locus on the short arm of chromosome 1A (Effertz et al. 2002).

ToxB is a small protein that causes necrosis in sensitive wheat lines. It was first identified in the culture filtrate of race 5 *Ptr* isolates, as well as in combination with other effectors in races 6, 7 and 8 (Strelkov et al. 2002; Lamari et al. 2003). The *ToxB* gene encodes a mature protein of 64 amino acids (Martinez et al. 2001), although no functional motifs that might contribute to toxic activity have been identified to date (Ciuffetti et al. 2010). *ToxB* copy number variation has been shown to impact on tan spot susceptibility, with isolates containing increased *ToxB* copies linked to higher *ToxB* gene expression and protein production and the induction of increased symptoms in the host (Strelkov et al. 2002; Strelkov and Lamari 2003; Martinez et al. 2004). ToxB-mediated chlorosis is known to be light dependent (Strelkov et al. 1998), and ToxB has been shown to inhibit photosynthesis and to modify the wheat leaf proteome prior to the development of chlorosis (Kim et al. 2010). The major ToxB sensitivity locus *Tsc2* has previously been mapped to the short arm of chromosome 2B (Friesen and Faris 2004; Abeysekara et al. 2010), accounting for up to 69% of total phenotypic variation (Friesen and Faris 2004).

Although ToxB was first characterised more than 17 years ago (Martinez et al. 2001), relatively little systematic investigation of wheat variety sensitivities has been undertaken to date, and the relationship between ToxB sensitivity and disease susceptibility has not been well defined. In this study, we present information on ToxB sensitivity in a collection of over 470 European wheat varieties and undertake genetic mapping of ToxB sensitivity using a combination of an association mapping panel, and an eight parent multiparent advanced generation intercross (MAGIC) population. Based on genotypic data generated using a 90 k single nucleotide polymorphism (SNP) array, fine mapping of the ToxB sensitivity locus *Tsc2* allowed development of a closely linked SNP-based marker for wheat disease resistance research and breeding.

## Methods

### Wheat germplasm and genotypic data

Three sets of wheat germplasm were used to assess ToxB sensitivity and for genetic mapping. (1) Parents of wheat genetic mapping populations: the eight founders of the ‘NIAB Elite MAGIC’ population (Mackay et al. 2014), eight founders of the ‘BMW MAGIC’ population (Stadlmeier et al. 2018), and 14 additional founders of various bi-parental populations and key germplasm stocks from hexaploid and tetraploid wheat (Table 1). (2) A wheat association mapping (AM) panel consisting of 480 predominantly UK, French and German varieties drawn from historic collections and National Lists, encompassing varieties released between 1916 and 2007 (Downie et al. 2018; Supplementary Table 1). (3) The ‘NIAB Elite MAGIC’ population: eight founders and 643 progeny. The AM and ‘NIAB Elite MAGIC’ populations were previously genotyped using an Illumina iSelect 90,000 feature single nucleotide polymorphism (SNP) wheat array (Wang et al. 2014). The AM panel data matrix consisted of 22,237 polymorphic SNPs (minor allele frequency ≥ 6%) across 480 varieties (data available at https://www.niab.com/pages/id/326/Resources), while the MAGIC data matrix consisted of 20,643 polymorphic SNPs across 643 progeny (Mackay et al. 2014; Gardner et al. 2016).

**Table 1:** Mean ToxB sensitivities for 29 wheat varieties

Variety	Populations and resources	Mean ToxB sensitivity
Alchemy	NIAB Elite MAGIC^1^	0.1
Ambition	BMW MAGIC^2^	0
Apogee	Apogee × Paragon^3^	0
Avalon	Avalon × Cadenza^4^. Avalon × Cadenza NILs^5^	0
Batallion	Oakley × Batallion^6^	2
BAYP4535	BMW MAGIC^2^	0
Brompton	NIAB Elite MAGIC^1^	0
Bussard	BMW MAGIC^2^	0
Cadenza	Avalon × Cadenza^4^. Avalon × Cadenza NILs^5^. TILLING^7^	0.2
CS	Paragon × CS^5^. Reference genome^8^	0
Claire	NIAB Elite MAGIC^1^. Claire × Malacca^9^. Genome sequence^10^	0.2
Dic12b^†^	Tios × Dic12b^11^	0
Event	BMW MAGIC^2^	0
Exsept	Oakley × Exsept^6^	0
Firl3565	BMW MAGIC^2^	0
Format	BMW MAGIC^2^	0
Gatsby	Oakley × Gatsby^6^	0
Hereward	NIAB Elite MAGIC^1^	0.2
Julius	BMW MAGIC^2^	0
Malacca	Claire × Malacca^9^	0
Oakley	Oakley × Batallion^6^, Oakley × Exsept^6^, Oakley × Gatsby^6^	0.1
Paragon	Paragon × CS^5^, Paragon × SHW CSSL^12^, genome sequence^10^	0
Potenzial	BMW MAGIC^2^	0
Rialto	NIAB Elite MAGIC^1^	0.2
Robigus	NIAB Elite MAGIC^1^. Genome sequence^10^	0
SHW-041	Paragon × SHW CSSLs^11^	0
Soissons	NIAB Elite MAGIC^1^	0.7
Tios^†^	Tios × Dic12b^11^	0
Xi19	NIAB Elite MAGIC^1^. Solstice × Xi19^9^	2.8

Sensitivity was scored on a 0 (no sensitivity) to 4 (highly necrotic) scale (See et al. 2019), with five replicates per genotype. The relevant major resources associated with each variety are listed. CS = cv. ‘Chinese Spring’. All varieties are hexaploid (*T. aestivum*), apart from those accessions indicated: ^†^tetraploid *T. turgidum* subsp. *dicoccum*. Resources listed are bi-parental populations, unless otherwise indicated. CSSL = chromosome segment substitution lines. NILs = near isogenic lines. References: 1 = Mackay et al. 2014. 2 = Stadlmeir et al. 2018. 3 = Allen et al. 2017. 4 = developed by C. Ellerbrook, L. Sayers, and T. Worland (John Innes Centre, UK). 5 = developed within the Wheat Genetic Improvement Network (WGIN) project, https://www.wgin.org.uk/. 6 = ERYCC report, available at https://cereals.ahdb.org.uk/media/200035/pr496.pdf. 7 = Krasileva et al. 2017. 8 = IWGSC RefSeq v1.0, available at https://wheat-urgi.versailles.inra.fr/Seq-Repository/Assemblies. 9 = Breeder’s population. 10 = available at https://wheatis.tgac.ac.uk/grassroots-portal/blast. 11 = developed within the Biotechnology and Biological Sciences Research Council (BBSRC) project, ‘WISP’ (BBSRC grant reference BB/I002561/1)

### ToxB effector production

ToxB protein was heterologously expressed via an *Escherichia coli* SHuffle strain and purified using immobilised metal affinity chromatography (IMAC) as described in See et al. (2019). Briefly, the DNA sequence that encodes the mature ToxB protein (GenBank accession PZD27634) (Martinez et al. 2001) was cloned into the pET21a(+) (Novagen) vector using the primers ToxB-sigP_F (5′-GGAATTCCATATGAACTGCGTCGCCAATAT-3′) and ToxB_R (5′-CCGCTCGAGACAACGTCCTCCACTTTGCA-3′) that included the engineered *NdeI* and *XhoI* sites (underlined), respectively. The resulting construct contained the predicted ToxB protein fused to a poly-histidine tag at the C-terminal, and was used to transform the *E. coli* SHuffle strain. The SHuffle strain harbouring the ToxB expression construct was grown in Terrific Broth medium and induced with β-D-1-thiogalactopyranoside (IPTG) at a final concentration of 100 µM for the expression of ToxB. Cells were harvested by centrifugation and resuspended in 5 ml binding buffer (20 mM sodium phosphate, 40 mM imidazole, 500 mM NaCl, pH 7.4) for the purpose of downstream his-tag purification. Cells were then lysed using sonication of 10-s pulses with 10-s intervals for 1 min (Sonoplus HD 3100, Bandelin, Germany). The cell debris was pelleted by centrifugation, and the ToxB protein was purified from the cell extract using HisPur Ni-NTA purification spin columns (Thermo Scientific) by gravity flow according to manufacturer’s instructions. Purified protein was dialysed in 20 mM sodium phosphate buffer, pH 7.4, quantified via the bicinchoninic acid assay (Smith et al. 1985) and stored in freeze-dried form at − 80 °C. Prior to infiltration, protein was resuspended in sterile water to a concentration of 200 µg/ml.

### Experimental design

All germplasm was sown into 96-well trays filled with M2 compost (Levington, Everiss). The experimental plan for each germplasm set comprised five replicates of each variety or accession, arrayed in a randomised block design using R/blocksdesign (cran.r-project.org). Seeds of the first germplasm set were grown in a growth chamber (Conviron) using a 16 h light (at 20 °C) and 8 h dark (15 °C) photoperiod. The AM and MAGIC populations were grown in a heated glasshouse (16 h light at 20 °C, 8 h dark at 17 °C) for 14 days with supplementary lighting to maintain photoperiod. ToxB infiltration was undertaken 14 days after sowing, following the method described by Moffat et al. (2014). Briefly, the first leaf of seedlings at growth stage 12 (GS12, Zadoks et al. 1974) was infiltrated with 50 µl of ToxB suspension at a concentration of 200 µg/mL, and the extent of infiltration along the leaf marked using a non-toxic pen. Phenotyping of ToxB sensitivity was carried out a week after infiltration and scored using a 0–5 scale, as described by See et al. (2019). A score of 0 = no visible symptoms; 1 = slight chlorosis; 2 = full chlorosis; 3 = extensive chlorosis with/without slight necrosis; 4 = chlorosis with necrosis; 5 = full necrosis. A water control was also used to establish a symptom baseline for the evaluation of possible damage during the infiltration process.

### Statistical analyses and QTL mapping

Summary statistics (mean, median, standard deviation and variance) were calculated using the software GenStat (VSN International, 16th edition). Best linear unbiased estimates (BLUEs) were calculated using a linear mixed approach in REML using GenStat (VSN International 2015). Heritability was calculated using GenStat: Broad sense heritability of line means was calculated from the estimate of the variance components in REML, taking into account all features of the experimental designs. Heritability was then estimated as *h*^2^ = *σ*^2^*G*/ (*σ*^2^*G* + *σ*^2^*e*) where *σ*^2^*G* is the genetic variance of line means and *σ*^2^*e* is the residual variance.

Genome-wide association scans (GWAS) using the AM panel were undertaken using the Efficient Mixed-Model Association algorithm (Kang et al. 2008) using a compressed mixed linear model that includes both fixed and random effects (Zhang et al. 2010), implemented with the Genome Association and Prediction Integrated Tool (GAPIT) package (Lipka et al. 2012) in R (R Core Team 2013). Genetic stratification in the population was corrected for using a kinship matrix constructed in GAPIT derived from a subset of SNPs skimmed from the complete set using an *r*^2^ threshold > 0.75. Further GWAS analysis was undertaken using selected markers as co-factors in the analysis, following the guidelines in the GAPIT manual. A Bonferroni-adjusted *P* = 0.01 (−log_10_*P* = 6.35) significance threshold was used.

MAGIC QTL mapping was carried out using 7369 unique mapped SNPs, with genetic map positions as described by Gardner et al. (2016). Four QTL analysis approaches were used: (1) SMA (single marker analysis): regression against single markers using R/lme4. (2) IBD (identity by descent): regression against haplotype probability estimates calculated using the ‘mpprob’ function in R/mpMap (Huang and George 2011) implemented in R/qtl (Broman et al. 2003) with a threshold of 0.5. (3) IM (interval mapping): conducted in R/mpMap using R/mpMap haplotype probability estimates. (4) CIM (composite interval mapping): conducted in R/mpMap with 5 or 10 covariates using R/mpMap haplotype probability estimates. For IBS and IBD analyses, multiple-test correction was carried out using *R*/*q* value, with a threshold of *q* < 0.05. For IM/CIM an empirical *P* threshold of 0.05 was determined for QTL analyses by conducting 100 simulations, using the sim.sigthr function in *R*. This value, together with a window size of 100 markers was used to determine QTL peaks using ‘find.qtl’. ‘Fit.qtl’ was then applied, and QTL retained which had *P* < 0.05 in the fitted model, as well as percentage variation explained > 1%.

### SNP anchoring and pedigree analyses

Selected SNPs were anchored to the wheat cv. Chinese Spring 42 IWGSC RefSeq v1.0 physical map (IWGSC 2018) by BLASTn (Altschul et al. 1990). Where BLASTn hits of equal match were identified on multiple chromosomes, genetic map position (Gardner et al. 2016) was used to assign hits to chromosomes. Wheat pedigree information was obtained from Fragley et al. (2019), with the underlying data available at https://www.niab.com/pages/id/326/Resources). The pedigree was displayed using Helium v.1.17.08.14 (Shaw et al. 2014) and images prepared using CorelDRAW (Corel Corporation, Canada).

### KASP genotyping

To validate the conversion of selected SNPs from the Illumina 90 k array to the Kompetitive Allele-Specific PCR (KASP) genotyping platform (LGC Genomics, UK), DNAs were extracted from the eight MAGIC founder varieties. Marker co-dominance was investigated using a 50:50 mix of DNAs from two varieties known from the 90 k SNP dataset to contrast for allele call. Additionally, DNA was extracted from a panel of 48 UK varieties that post-date the AM panel, released to the AHDB Recommended List between 2009 and 2017 (Supplementary Table 2). All DNAs were extracted from two week old leaves using a modified Tanksley protocol (Fulton et al. 1995), and concentration was determined using a Nanodrop 200 spectrophotometer (Thermo Scientific). DNA sequences flanking each targeted SNP were used to design KASP primers using the software PolyMarker (Ramirez-Gonzalez et al. 2015). Primers were synthesised by Sigma-Aldrich (Cambridge, UK) and KASP genotyping undertaken as described by Cockram et al. (2015). The results were visualised using SNP Viewer v.1.99 (https://lgcgenomics.com/). The subset of plants that post-dated the AM panel were grown and phenotyped for ToxB sensitivity and genotyped with the KASP marker, following the methods described above.

### Genomics analyses of the Tsc2 region of synthetic wheat line W7984

Analysis of the *Tsc2* region was carried out in eleven steps. (1) The W7984 assembly scaffolds (Chapman et al. 2015) were aligned to the reference ‘Chinese Spring’ genome, RefSeq v1.0 (IWGSC 2018) with MiniMap2 v2.15 (Li 2018) using command line options: ‘-I 20G -ax asm5 –secondary = no’. Scaffolds with primary alignments to a region conservatively encompassing the *Tsc2* locus (2B: 21536610..0.27030113 bp) (SAM flags 0 and 16) were extracted from the W7984 assembly and ordered and orientated based on their SAM file coordinates and mapping orientation flags before being joined together as one fragment with each contributing scaffold separated by 100 Ns. (2) The synthetic 2B assembled fragment and the corresponding region of CS42 (chr2B: 21536610..0.27030113 bp) were then repeat masked using Repeatmasker v4.0.8 (https://www.repeatmasker.org) using the command line option: ‘-species Triticum aestivum’. (3) The two repeat masked sequences were aligned using minimap2 v2.16, using the ‘Chinese Spring’ region as reference and with the command line options:‘–cs = long -c -t 20 -K 500 M –Y’. Methods for the liftover of the ‘Chinese Spring’ gene models are further described in Supplementary Text 1. (4) RNA-seq reads (> 8 billion) from BioProject accession numbers PRJDB2496, PRJEB12497, PRJEB25593, PRJEB25639, PRJEB25640, PRJNA213168, PRJNA243835 were obtained from the National Center for Biotechnology Information (NCBI) Short Read Archive (SRA). Further details on the processing of the RNA-seq data and subsequent transcript assembly, gene model and splice junction prediction are listed in Supplementary Text 1. (5) Protein datasets sourced from the genome assemblies of three cereal species (listed in Supplementary Text 1) were aligned to our synthetic 2B fragment from ‘W7984’ that spanned the *Tsc2* region using GenomeThreader v1.7.1 (Gremme et al. 2005). Protein alignments were further filtered using the script ‘filter_exonerate.py’ from the ei-annotation suite (https://github.com/lucventurini/ei-annotation), using the Portcullis junctions above as externally validated junctions and with further parameters: ‘-minI 20 -maxE 1000 -maxM 5000’, which excluded any hit with terminal introns longer than 1 kbps or internal introns longer than 5 kbps. (6) Transcript reconstruction: Mikado (Venturini et al. 2018) leverages transcript assemblies generated by multiple methods to improve transcript reconstruction. Loci were first defined across all input assemblies with each assembled transcript scored based on metrics relating to open reading frame (ORF) and cDNA size, relative position of the ORF within the transcript, un-transcribed region (UTR) length and presence of multiple ORFs. Mikado was used to integrate the Illumina assemblies generated above, as described in further detail in Supplementary Text 1. (7) Gene predictions: the information obtained from steps 1–6 was used for final gene prediction using Augustus (Stanke and Morgenstern 2005) as described in Supplementary Text 1. (8) Determining the likelihood of gene presence/absence in ‘Chinese Spring’: Percentage gene presence in ‘Chinese Spring’ (CS) was determined by using BLAST to query the CS chr2B region for each CDS followed by calculating the mean average of the best % CDS presence (nident/qlen * 100) for each transcript. Where there was more than one splice variant present, the transcript with the highest average CDS presence was used to represent the % gene presence in CS. (9) Functional annotation of the synthetic 2B assembled fragment predicted genes was carried out via BLAST queries to the uniprot90 protein database (releaseDate = ‘2019-04-10’ version = ‘2019_03’). For each predicted peptide, the top 20 BLAST hits (based on e-values ≤ 1e−25) were retained. The gene functions were then assigned by hand. Where there was no function known or where BLAST results were > 1e−25, proteins were labelled as uncharacterised. (10) Identifying the corresponding CS gene IDs to the synthetic gene predictions: The coding sequences for each of the transcripts from both the synthetic region and corresponding CS region were extracted using gffread. BLAST was used to query the CS coding sequences with the predicted synthetic coding sequences. The best BLAST results (determined by e-value and bit score) were taken for each query and coding sequences with less than a 90% CDS identity (nident / qlen * 100) were ignored. (11) Circos (Krzywinski et al. 2009) plots were constructed using a 10-kb jumping window for both gene and repeat counts. Marker positions within the synthetic assembled region were determined using BLAST. Canonical kmer repeats (> 1) are 91 bp and contain no *N* labelled bases. Candidate genes were identified using the following search terms from the Uniprot functional annotation 'resistance', 'defence' 'disease', 'wall-associated', 'lrr', 'nbs', 'stress' and 'cysteine-rich'.

## Results

### ToxB sensitivity in a panel of wheat genetic mapping population founders

ToxB infiltration of the panel of 29 parental lines of wheat mapping populations found moderate to high sensitivity scores (≥ 2) in just two accessions (Table 1). All eight founders of the ‘BMW MAGIC’ population were insensitive (score < 0.2) to ToxB, ruling out this population for QTL analysis. Of the eight ‘NIAB Elite MAGIC’ founders, six lines were insensitive (score ≤ 0.2; ‘Alchemy’, ‘Brompton’, ‘Claire’, ‘Hereward’, ‘Rialto’, ‘Robigus’) one showed intermediate sensitivity (‘Soissons’, score = 0.7), while one was highly sensitive (‘Xi19’, score = 2.8). Among the remaining 12 varieties tested, only ‘Battalion’ showed marked ToxB sensitivity (score = 2), with all other lines classified as ToxB insensitive (score ≤ 0.2; ‘Cadenza’, ‘Oakley’, ‘Apogee’, ‘Avalon’, ‘Chinese Spring’, ‘Dic12b’, ‘Exsept’, ‘Gatsby’, ‘Malacca’, ‘Paragon’, ‘SHW-041’ and ‘Tios’). Based on these results, two wheat populations were selected for further analysis: ‘NIAB Elite MAGIC’, and a European AM panel so as to screen as widely as practicable for the evidently rare cases of sensitivity.

### Genetic analysis of ToxB sensitivity in the MAGIC and AM populations

**Fig. 1:**
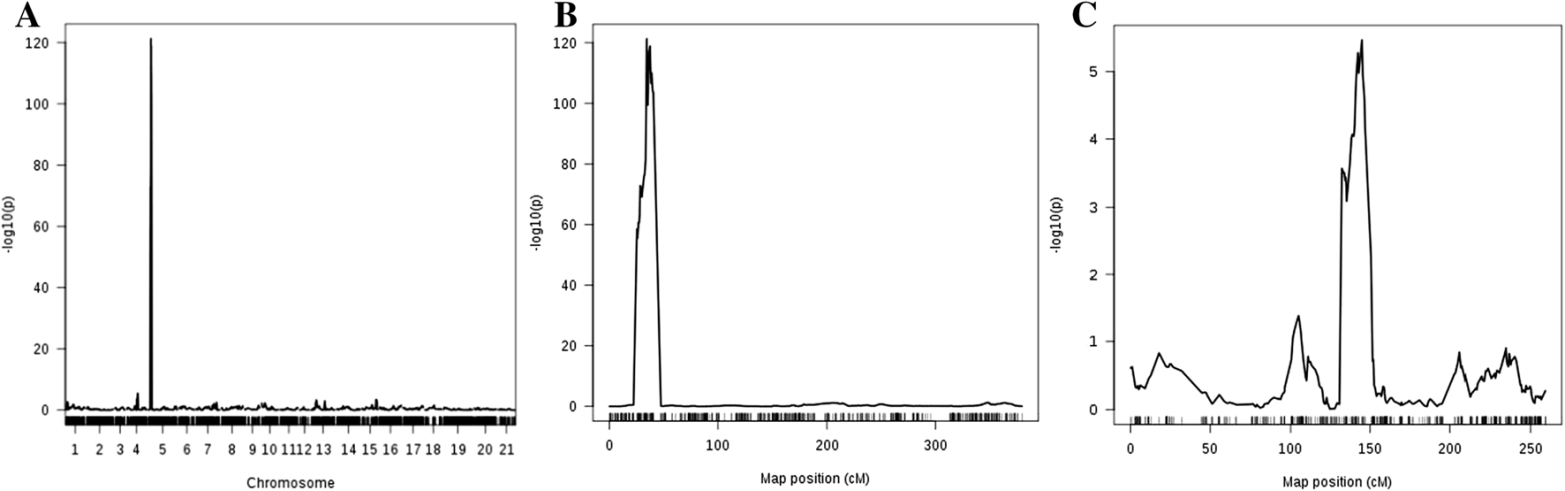
An example of the results of genetic analysis of ToxB sensitivity in the ‘NIAB Elite MAGIC’ population. Presented here are the outputs using composite interval mapping (CIM), covariates = 10. (A) QTLs found across all 21 wheat chromosomes with 1 (1A), 2 (1B), 3 (1D), 4 (2A) through to 21 (7D). (B) Chromosome 2B, showing the major ToxB sensitivity locus, *Tsc2*. (C) Chromosome 2A, showing the putative minor ToxB sensitivity QTL located at ~ 142 cM

ToxB sensitivity in the MAGIC population (*n* = 643) was found to be highly heritable (*h*^2^ = 0.95). Mean ToxB sensitivity in the progeny ranged between 0 (insensitive) and 2.25, with 503 lines = 0, 70 lines > 0 ≤ 1 and 67 lines > 1. QTL mapping of ToxB sensitivity in the MAGIC population using log_10_ transformed phenotypic data and four genetic mapping approaches (SMA, IBD, IM, CIM) all identified a major QTL on the short arm of chromosome 2B (e.g. for IM analysis, −log_10_*P* = 109.19, accounting for 41% of the phenotypic variation) (Fig. 1; Table 2; Supplementary Table 1). To help determine the *Tsc2* interval, CIM using 5 and 10 covariates and an empirical *P* = 0.01 significance threshold (−log_10_*P* = 5.29) identified a 15.11 cM region (from SNPs BS00034887_51 to BS00053520_51). Within this region, via systematic comparison of the *P* values along with the genetic and physical map positions of the markers, we define the peak of this QTL to be located in a 1.094 Mbp interval from 24.092 Mbp (BS00070051_51) to 25.186 Mbp (BS00002660_51), which includes all markers with −log_10_*P* > 90 from the IM and CIM analyses. This locus corresponds to be the major ToxB sensitivity locus *Tsc2* (Friesen and Farris 2004), based on the position of the MAGIC SNPs identified here and the previously identified *Tsc2*-linked flanking markers *TC339813* and *BE444541* (Abeysekara et al. 2010), which we find to be located on chromosome 2B at 22.230 and 24.100 Mb, respectively. Additionally, a putative minor QTL was identified on the long arm of chromosome 2A, identified using CIM-cov10 (−log_10_*P* = 5.70) (Fig. 1; Table 2). This QTL is located between 132.174 and 146.829 cM with the QTL peak defined by SNP markers Kukri_c24064_2095 and BS00022641_51 and explained 1.4% of the phenotypic variation. Given the locations of the 2A and 2B QTLs on the long and short chromosomal arms, respectively, these are not assumed to be homoeologous loci. Note that for the minor 2A QTL, while the CIM-cov10 result was above the empirical *P* = 0.05 (–log_10_*P* = 4.36) and *P* = 0.01 (−log_10_*P* = 5.29) significance thresholds, using CIM-cov5 it was found to be insignificant (−log_10_*P* = 3.68).

**Table 2:** Summary of the QTL identified for ToxB sensitivity using the ‘NIAB Elite MAGIC’ population

Chr	Left marker name (IWGSC RefSeq v1.0 Mb position)	Right marker name (IWGSC RefSeq v1.0 Mb position)	−log_10_*P*	PVE (%)
2B	BS00070050_51 (24.09)	Kukri_c63748_1453 (25.02)	109.19	41.0
2A	CAP12_rep_c7918_56 (613.00)	RAC875_c38018_278 (639.99)	5.70	1.4

The chromosome 2B QTL, *Tsc2*, was identified with all mapping methods tested: IM (data presented in the table above) and CIM using 5 and 10 covariates, IBS and IBD, with the best QTL resolution obtained with CIM-cov5 and -cov10. The 2A QTL was identified using CIM-cov10 (data presented in the table) but was not significant at CIM-cov5 (−log_10_*P* = 3.68) using the empirical *P* = 0.05 threshold. Flanking markers listed represent those immediately adjacent to the peak of the QTL, based on genetic map order. Chr = chromosome. PVE = phenotypic variation explained. Further details for the markers with all QTL intervals are listed in Supplementary Table 1

**Fig. 2:**
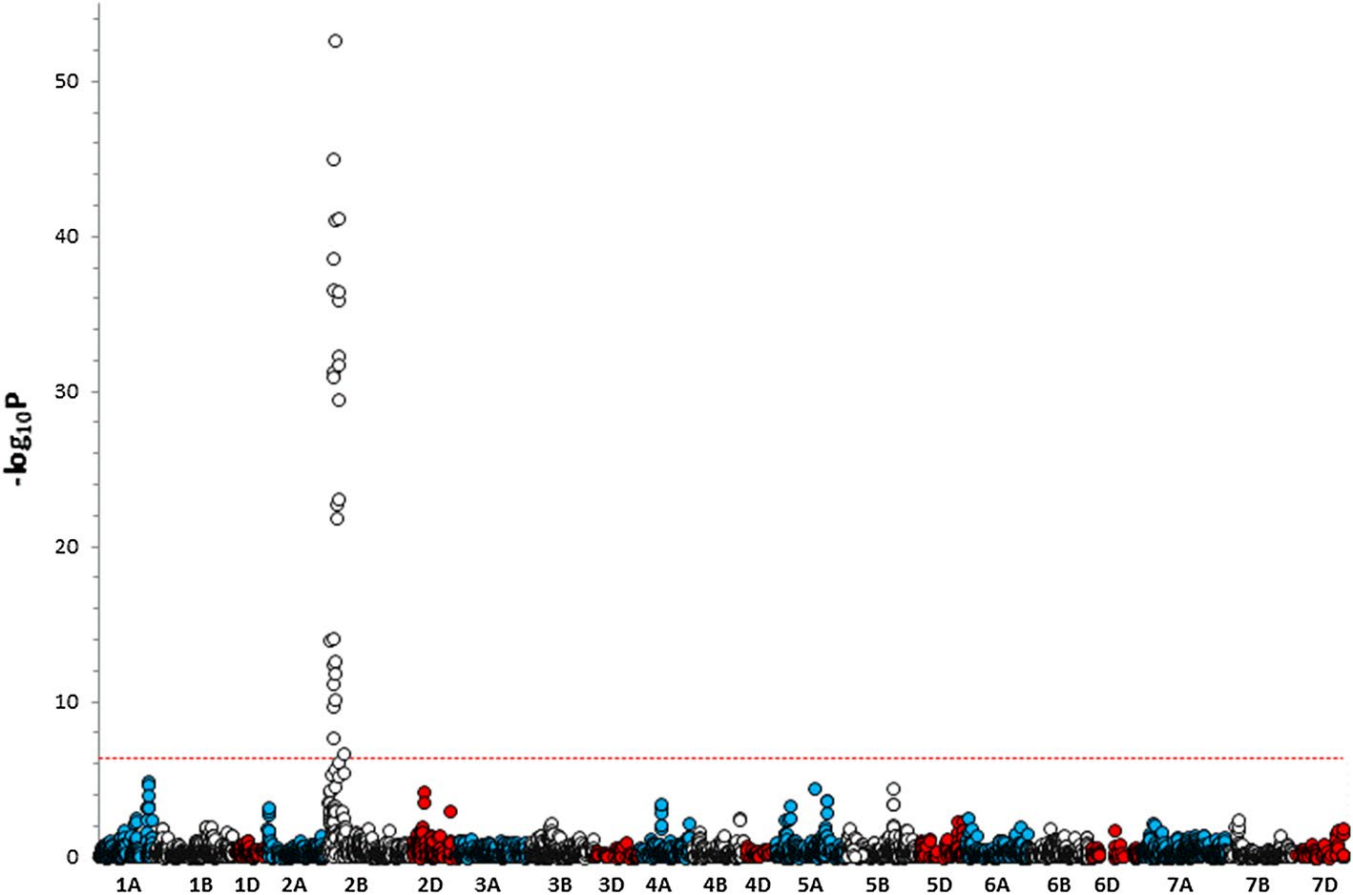
Manhattan plot of GWAS results for ToxB sensitivity in the association mapping panel (*n* = 480) using 22,237 SNPs. Markers are ordered according to the ‘NIAB Elite MAGIC’ genetic map (Gardner et al. 2016). The Bonferroni-adjusted *P* = 0.01 significance threshold (−log_10_*P* > 6.35) is indicated. The 21 wheat chromosomes, 1A through to 7D, are indicated. Unmapped markers are not shown

ToxB sensitivity was also found to be highly heritable in the AM panel (*n* = 480, *h*^2^ = 0.9). The majority of varieties (93%) were found to either be insensitive (ToxB sensitivity < 0.2) or slightly sensitive (sensitivity > 0.2, < 1), while just 7% of varieties were classified as sensitive (≥ 1) (Supplementary Table 2). GWAS using the 22,237 SNPs after skimming the complete data matrix on minor allele frequency ≤ 0.06 identified 73 significant SNPs at the Bonferroni corrected *P* = 0.01 threshold (−log_10_*P* > 6.35) (Fig. 2; Supplementary Table 3). Of these SNPs, 64 were located on the MAGIC genetic map on chromosome 2B between 15.13 and 73.65 cM. Anchoring the remaining nine SNPs to the physical map via BLASTn indicated eight mapped to chromosome 2B (Supplementary Table 3) and supported by the analyses of Gardner et al. (2016) who treated these SNPs as a trait and QTL mapped them back to chromosome 2B on the ‘NIAB Elite MAGIC’ genetic map. For the remaining unmapped SNP (Kukri_c30668_294), while BLASTn predicted it to map to 2D, using the SNP as a trait predicted it to be located on 2B (Gardner et al. 2016). Accordingly, we tentatively assign this marker to chromosome 2B.

Investigation of the haplotypes defined by the 12 SNPs genotyped across the peak of the *Tsc2* locus in the AM panel allowed a condensed five-SNP haplotype across the region to be determined (Supplementary Table 4). This defined 10 haplotypes, six of which were associated with low ToxB sensitivity (the ‘group 1’ haplotypes: 1.1 to 1.6) and four with high sensitivity (the ‘group 2’ haplotypes: 2.1 to 2.4), with the differences in ToxB sensitivity between these two haplotype groups found to be highly significant (Welch two sample *t*-test: *t* = 25.5, *P* ~ 0) (Supplementary Figure 1). Additionally, clear recombinations proximal (haplotype 2.2) and distal (haplotypes 2.3 and 2.4) to *Tsc2* delimited the target region to a 1921-kb region between 231.063 Mbp (TraesCS2B01G046400) and 250.180 Mbp (TraesCS2B01G051000) in the AM panel. All three of the most significant SNPs identified by GWAS (−log_10_*P* = 52.63; BS00070050_51, BS00072620_51 and BS00075303_51) originated from gene model TraesCS2B01G048500, and all three cosegregate on the ‘NIAB Elite MAGIC’ genetic map at 34.38 cM. Additionally, BS00070050_51 was also identified as the marker immediately proximal to the *Tsc2* QTL peak in the MAGIC IM and CIM analyses. In the AM panel, this SNP perfectly predicts sensitivity to ToxB, with alleles A:A and G:G diagnostic for sensitivity (score ≥ 1) and insensitivity (score ≤ 0.6), respectively (Table 3; Supplementary Table 2). When GWAS was repeated using the *Tsc2*-lined marker BS00070050_51 as a cofactor, no additional significant associations were found.

**Table 3:** Comparison of allele scores at the *Tsc2*-linked SNP marker BS00072620_51 with the number of varieties in the AM panel found to have low and high ToxB sensitivity (sensitivity scores < 1 and ≥ 1 ≤ 3, respectively)

BS00072620_51 genotype	ToxB sensitivity < 1	ToxB sensitivity ≥ 1	Totals
A:A	442	0	442
B:B	0	38	38
	442	38	480

The 480 accessions used represent those with both genotype and phenotype data

### Bioinformatic analysis of the Tsc2 region

In summary, the genetic analyses defined a 1921-kb region defined in the AM panel (23.106 to 25.027 Mbp), extended proximally to 25.236 Mbp by the proximal end of the MAGIC peak region. This combined interval of 2129 kb contains 108 predicted genes in the ToxB-insensitive variety ‘Chinese Spring’ (Supplementary Table 5). Within the interval, the most significant SNPs from the AM panel and the MAGIC population were all located within gene model TraesCS2B01G048500, a glyoxylate reductase/hydroxypyruvate reductase located at 24.092 Mbp. Additional SNPs identified in the AM panel were contained within the interval: hit 6 (GENE-1343_556) originates from TraesCS2B01G048700 which encodes an arginase while hit 12 (Kukri_c63748_1453) is predicted to encode a cullin-associated NEDD8-dissociated protein 1. The wheat gene controlling sensitivity to the *Ptr* effector ToxA encodes a protein kinase NBS-LRR protein (Faris et al. 2010). A gene model predicted to encode an NBS-LRR protein is located within the 2129-kb ‘Chinese Spring’ region identified here (TraesCS2B01G050500). However, TraesCS2B01G050500 does not show high sequence similarity to *Tsn1*. Additionally, gene model TraesCS2B01G051200 annotated as an *MLO-*like gene, was located towards the distal end of the MAGIC QTL peak. Several additional disease resistance pathway genes were located just outside the *Tsc2* interval, including an *NBS-LRR* gene from which hits 9 and 13 originated (TraesCS2B01G045700), and surrounded by a wider cluster of seven additional NBS-LRR genes.

**Fig. 3:**
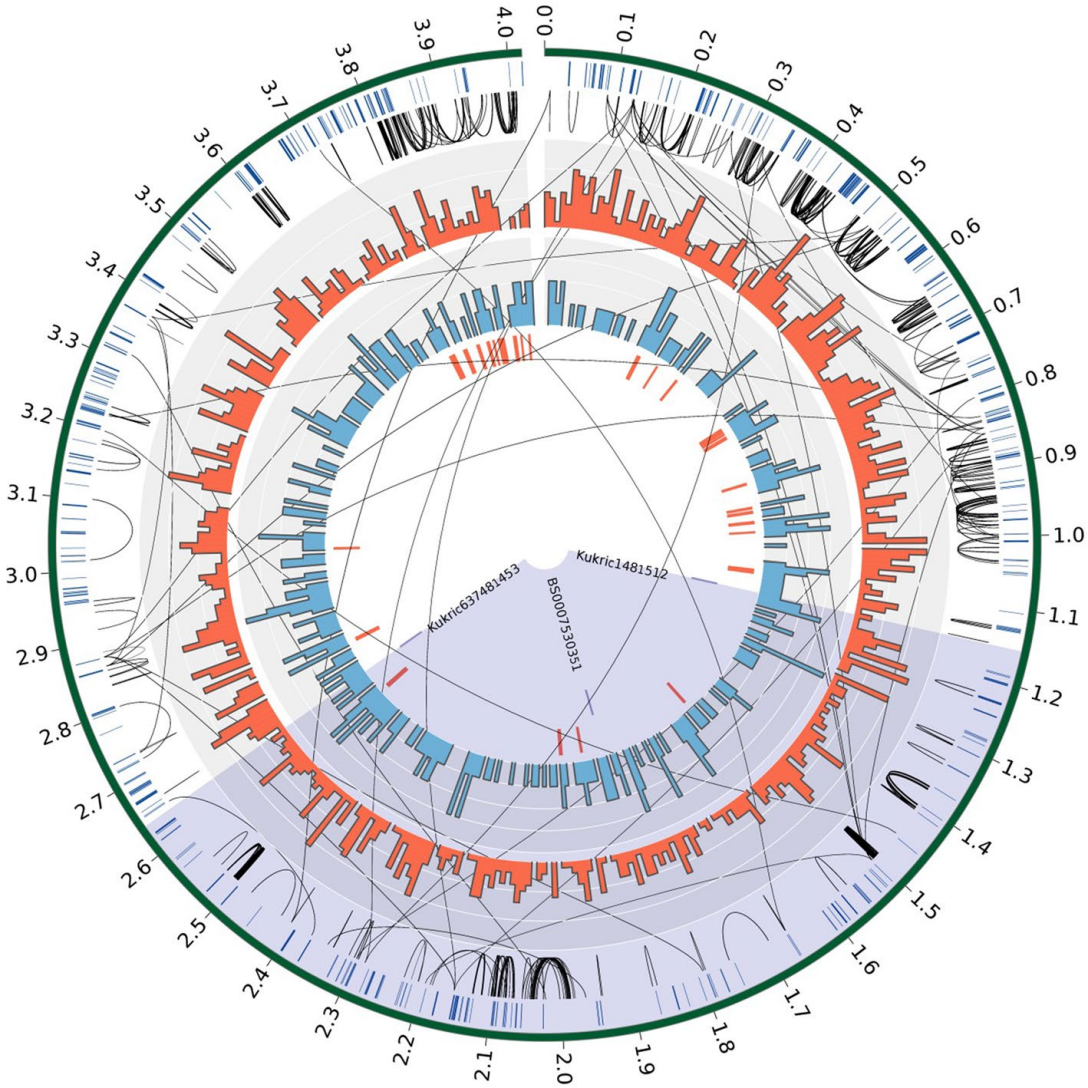
Circos plot illustrating key genomic and genic features of the ‘Synthetic W7984’ ‘super-contig’ (705 assembly scaffolds, totalling 3.47 Mb) spanning the wider *Tsc2* locus, based on the ‘Chinese Spring’ region 2B: 14,040,000–30,500,000 bp. Tracks, from outside to inside: (1) the super-contig, with size indicated in Mb. (2) tick marks to illustrate start/end points between scaffolds, (3) loops to indicate sequence homology of genes based on 91-mers, (4) histogram of transposable element density, (5) histogram of gene density, (6) candidate genes, (7) tick marks indicating the named SNPs that delineate the boundaries of the most likely *Tsc2* region: (a) regions kukric1481512 to kukric637481453 (full names Kukri_c148_1512 and Kukri_c63748_1453, respectively) represent a conservative *Tsc2* interval, based on clear recombinations in the AM panel and encompassing the peak of the MAGIC QTL in this region, (b) region BS0007530351 (full name BS00075303_51, originating from the same RefSeqv1.1 gene model as BS00070050_51) to kukric637481453, which spans the peak of the MAGIC QTL and contains AM panel GWAS hits 1–6, 10 and 12

‘Chinese Spring’, for which the wheat reference genome is available, is insensitive to ToxB. Based on this, as well as the existing evidence in the published literature and the identification of a single ToxB sensitivity locus via analysis of the association mapping panel undertaken here, Chinese Spring would be expected to contain a non-functional allele/deleted gene at the *Tsc2* locus. Of the few additional hexaploid wheat varieties for which a genome sequence is currently available, only one has previously been shown to be ToxB sensitive: ‘W9784’ (Friesen and Faris 2004). This is a synthetic hexaploid wheat, derived by the hybridisation of the tetraploid wheat species *Triticum durum* Desf. with the diploid wheat progenitor *Aegilops tauschii*. Although the contig length of the ‘W9784’ de novo genome assembly (Chapman et al. 2015) represents just 55% (7.88 Gb) of the 14.27 Gb ‘Chinese Spring’ RefSeq v1.0 assembly (IWGSC 2018), we nevertheless attempted to exploit this resource to investigate possible gene content in the *Tsc2* region. We first aligned the ‘W9784’ assembly to the ‘Chinese Spring’ genome, and then extracted primary alignments to a highly conservative physical interval across the ‘Chinese Spring’ *Tsc2* region (2B: 14,040,000–30,500,000 bp), spanning GWAS hit 15 (BS00085748_51, proximal) through to hit 23 (BS00022572_51, distal). This region comfortably includes the 2129-kb interval as defined from the combined AM and MAGIC analyses. The ‘W9784’ scaffolds were stitched together to form a single ‘super-contig’ (Fig. 3) consisting of 705 scaffolds with a mean scaffold size of 5.6 Kb. The un-gapped assembled super-contig was 3.47 Mb representing 64.3% of the ‘Chinese Spring’ region (5.39 Mb). We then undertook gene annotation of the ‘W9784’ super-contig (see Supplementary Text 1), resulting in the identification of 379 gene models (Supplementary Table 6). After excluding gene models annotated as transposable elements, 23 potential candidate genes were identified in the remaining 347 models, including four within the GWAS interval encompassing hits 1–8, 10 and 12 (from SNPs Kukri_c148_1512 to Kukri_c63748_1453; ‘W9784’ gene models jg131, jg166 and jg172) (Supplementary Table 6). Three of these candidates were annotated as LRR receptor-like serine/threonine protein kinases. Gene jg131 is collinear with ‘Chinese Spring’ gene model TraesCS2B01G047800, while jg166 is absent in the collinear region of ‘Chinese Spring’, and jg172 appears to be present (99% average CDS nucleotide identity), but not annotated as a gene in ‘Chinese Spring’. In addition, a WRKY transcription factor (jg224), collinear with ‘Chinese Spring’ gene TraesCS2B01G050500 annotated as a TIR-NBS-LRR domain disease resistance protein, was identified. Of these, jg166, jg172 and jg224 were located within the interval defined by the peak of the MAGIC QTL (from SNPs Kukri_c148_1512 to Kukri_c63748_1453). Using a cut-off of 80% CDS sequence similarity, 51 genes in the ‘W9784’ assembly were found to be absent from the *Tsc2* region in ‘Chinese Spring’ (Supplementary Table 6). Details of all ‘W9784’ genes and candidate genes are summarised in Supplementary Table 6. As the contig size of the ‘W9784’ assembly is very low, with an N50 of 6.7 kb, we did not undertake further characterisation of its genic content. Nevertheless, this information provides a starting point with which to undertake future studies.

### Development of a diagnostic KASP marker linked to Tsc2

**Fig. 4:**
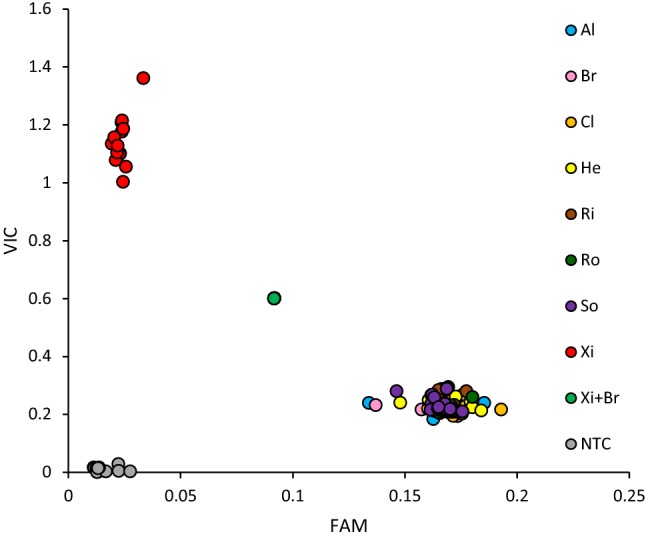
Allele calls for the *Tsc2-*linked KASP marker developed for SNP BS00072620_51. Fluorescence intensity detected for the FAM (representing nucleotide call G:G, associated with ToxB sensitivity, score ≥ 1) and VIC (A:A, ToxB insensitive, score < 1) fluorophores are shown on the x-axis and y-axis, respectively. Genotyping was undertaken using the DNA extracted from the ‘NIAB Elite MAGIC’ founders. Al = ‘Alchemy’, Br = ‘Brompton’, Cl = ‘Claire’, He = ‘Hereward’, Ri = ‘Rialto’, Ro = ‘Robigus’, So = ‘Soissons’, Xi = ‘Xi19’, Xi + Br = marker co-dominance tested by inclusion of a 50:50 mix of ‘Xi19’ and ‘Brompton’ DNA. NTC = no template negative control

The highly significant SNP BS00072620_51 identified in both the AM and MAGIC populations was selected for conversion from the 90 k SNP array to the KASP genotyping platform. The KASP primers designed (Allele-specific-A: 5′-tgattgcggagtatgtgca-3′, Allele-specific-B 5′- tgattgcggagtatgtgcg-3′, Common primer: 5′-gcaatgcgtgtccgtgtaaata-3′) were tested on DNAs extracted from all eight MAGIC founders, as well as a 50:50 mix of DNAs from ‘Brompton and ‘Xi19’, and found tight clustering of both homozygous allele classes, while robust calling of the artificially admixed DNA confirmed the KASP marker to be co-dominant (Fig. 4). The marker was subsequently used to genotype a collection of 47 elite UK winter wheat varieties that post-dated the AM panel, released on the UK AHDB Recommended List (https://cereals.ahdb.org.uk/varieties/ahdb-recommended-lists.aspx) between 2009 and 2017 (Supplementary Table 7). Of these, 39 were found to be A:A homozygous alleles, while 8 were G:G (predictive of ToxB insensitivity and sensitivity, respectively). Phenotyping these 47 recent varieties for ToxB sensitivity found the KASP allele call to correctly predict phenotype in all cases (Supplementary Table 7).

### Wheat pedigree analysis of ToxB sensitivity

**Fig. 5:**
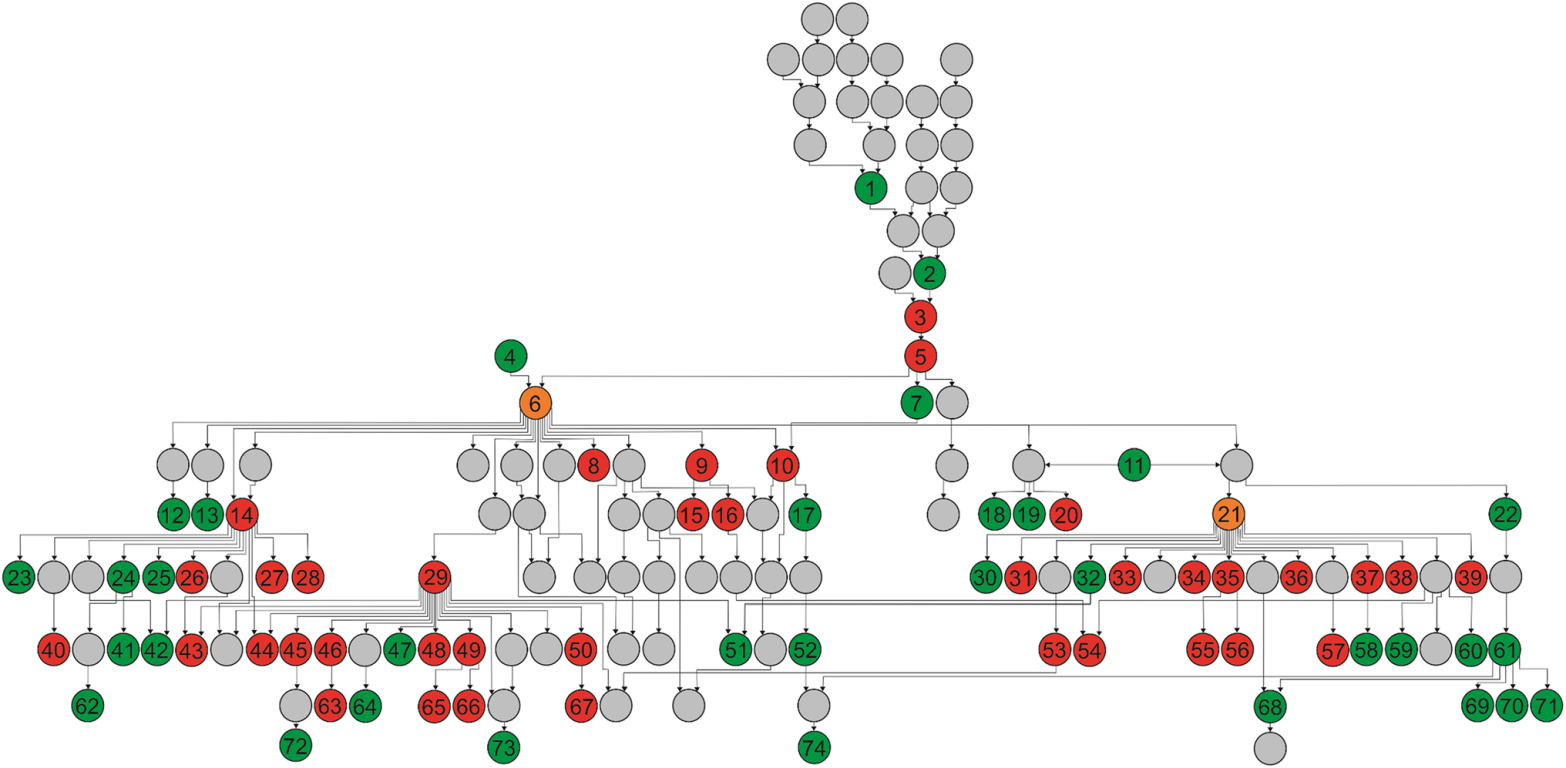
ToxB sensitivity in the context of wheat pedigree information. ToxB-sensitive (score ≥ 1) and insensitive (score < 1) lines are highlighted in red and green, respectively. Lines or steps in the parentage for which no ToxB phenotype data were available are indicated in grey. The varieties ‘Cadenza’ (node 6) (and its sibs) and ‘Aardvark’ (node 21) are highlighted in orange and are hypothesised here to have segregated for allelic status at the *Tsc2* locus during the early period they were used for breeding, with insensitive alleles having subsequently been fixed in commercially released germplasm for both lines. (1) Garnet, (2) Koga II, (3) Maris Dove, (4) Tonic, (5) Axona, (6) Cadenza, (7) Shiraz, (8) Warlock 24, (9) Scorpion 25, (10) Raffles, (11) Lynx, (12) Phlebas, (13) Convoy (14) Xi19, (15) Limerick, (16) Duxford, (17) Cantata (sib), (18) Brando, (19) Ashanti, (20) Arriva, (21) Aardvark, (22) Aardvark (sib), (23) Velocity, (24) Bantham, (25) Rocky, (26) Gallant, (27) Cocoon, (28) Walpole, (29) Cordiale, (30) Galtic, (31) Cadogan, (32) Gulliver, (33) Aarden, (34) Bowindo, (35) Battalion, (36) Scandia, (37) Hyperion, (38) Marksman, (39) Choice, (40) Panorama, (41) KWS Barrel, (42) KWS Podium, (43) Cubanita, (44) KWS Curlew, (45) Grafton, (46) KWS Quartz, (47) Acrobat, (48) Orbit, (49) KWS Sterling, (50) KWS Horizon, (51) Crusoe, (52) Ambrosia, (53) Dover, (54) Moulton, (55) Bennington, (56) RGT Illustrious, (57) Buzzer, (58) Gravitas, (59) Freiston, (60) Dunston, (61) Oakley, (62) LG Mowtown, (63) KWS Zyatt, (64) Costello, (65) KWS Siskin, (66) KWS Silverstone, (67) KWS Lilli, (68) KWS Santiago, (69) Reflection, (70) KWS Gator, (71) KWS Kielder, (72) KWS Trinity, (73) Ranger, (74) RGT Conversion. A high-resolution image in which the variety names for each node in the pedigree are included directly in the figure is available for download as a Supplementary Figure 2

Overlaying ToxB sensitivity scores from the AM panel and post-AM panel onto an up-to-date wheat pedigree (Fradgley et al. 2019) allowed the likely transmission of the ToxB-sensitive allele(s) to be tracked (Fig. 5; Supplementary Table 2). The oldest ToxB-sensitive accession identified was the spring variety ‘Thatcher’ (released in the USA in 1934), followed by ‘Aronde’ (France, 1962) and ‘Maris Dove’ (UK, 1971). Analysis of the pedigree found three varieties to act as ‘hubs’ for frequent transmission of ToxB sensitivity: (1) ‘Xi19’ (UK, year of release = 2000, sensitivity = 2.9) had six sensitive descendants (‘Cocoon’, ‘Cubanita’, ‘Curlew’, ‘Gallant’, ‘KWS Curlew’ and ‘Panorama’), collectively representing 15% of the sensitive European varieties identified. (2) ‘Cordiale’ (UK, 2001, sensitivity = 1.2) had 12 sensitive descendants, representing around a quarter of ToxB-sensitive accessions. (3) The winter wheat variety ‘Aardvark’ (UK, 1997), parent or grandparent of 12 sensitive varieties, representing 27% of all ToxB-sensitive lines identified. Despite its prominence in the pedigree of ToxB-sensitive varieties, ‘Aardvark’ was found to be insensitive. All three ‘hub’ varieties shared features in common: all were formally found to have ToxB-insensitive parents, and all had ‘Cadenza’ or a sib of ‘Cadenza’ listed as one of their parents (Fig. 5). It should be noted that ‘Maris Dove’ and its progeny ‘Axona’ are sensitive, and ‘Axona’ is a parent of ‘Cadenza’. Together, this indicated the most plausible transmission route of ToxB sensitivity to the hub varieties ‘Xi19’ and ‘Cordiale’ is via ‘Cadenza’ breeding lines/sibs still segregating at the *Tsc2* locus*.* As the remaining hub variety ‘Aardvark’ has ‘Cadenza sib’ as one of its parents, and where data were available, none of the remaining parents for the 12 ToxB-sensitive ‘Aardvark’ descendants were found to possess sensitivity, we speculate that ‘Aardvark’ germplasm used for breeding was also segregating at *Tsc2*. The assumption of segregation at *Tsc2* in ‘Cadenza’ and ‘Aardvark’ germplasm used during their early use in breeding results in all but eight of the 46 ToxB-sensitive varieties being linked in a single pedigree, within which ToxB sensitivity arose from ‘Maris Dove’.

## Discussion

### Sensitivity of north-western European wheat to ToxB

Despite the cloning of *ToxB* over 17 years ago (Martinez et al. 2001), relatively few screens for ToxB sensitivity in wheat have been published. Surveys of Canadian wheat varieties found sensitivity to be present in around 30% of wheat varieties (Tran et al. 2017: 24 of 100 cultivars. Lamari et al. 2005a: 30 of 86 cultivars), while in a recent screen of 122 Australian varieties just 4% were ToxB sensitive (See et al. 2019). Here, screening an AM panel of 480 varieties found ToxB sensitivity to be uncommon in the European germplasm screened, with just 7% possessing a sensitivity score ≥ 1. It appears that ToxB sensitivity was introduced via the variety ‘Thatcher’, released in the USA in 1931. ‘Thatcher’ was used 41 times as a parent in the pedigree, and was a parent of the second oldest sensitive variety in the AM panel, ‘Aronde’ (France, 1962). ‘Thatcher’ has been previously identified as the possible source of ToxB sensitivity in Canadian wheat (Lamari et al. 2005a; Tran et al. 2017), highlighting its prominent role in wheat pedigrees across the world.

Analysis of ToxB sensitivity within the context of the wheat pedigree found three varieties to represent hubs for frequent transmission of ToxB sensitivity: ‘Xi19’ (‘Cadenza’ × ‘Rialto’), ‘Cordiale’ (‘Cadenza’ × ‘Reaper’) and ‘Aardvark’ ([‘Cadenza sib’ × ‘Lynx’] × ‘Lynx’). However, a good indication was found that the germplasm for ‘Cadenza’ and its sibs used during early breeding activities segregated for alleles at *Tsc2*. This was based on the following evidence: (1) the inability to trace the source of sensitivity in the immediate parents of all three hub varieties, (2) the observation that all hub varieties contained ‘Cadenza’ in their immediate parentage, (3) the ToxB-sensitive variety ‘Arriva’ represents a ‘Lynx’ (insensitive) × ‘Cadenza’ cross, and (4) ‘Cadenza’ has the sensitive variety ‘Axona’ as one of its parents. A similar assumption was made for the key hub variety ‘Aardvark’, whose parentage includes a sib of ‘Cadenza’ × ‘Lynx’ (insensitive), and which ToxB phenotyping found to be insensitive, despite being a parent of 12 varieties for which no other source of sensitivity was found. On the assumption of segregation at the *Tsc2* locus for the ‘Cadenza’ and ‘Aardvark’ germplasm used during breeding, all but eight ToxB-sensitive lines could be traced via the pedigree to the spring wheat variety ‘Maris Dove’, released in the UK in 1971. Of the eight ToxB-sensitive lines not accounted for in the pedigree, one was a coded breeder’s line with unknown parentage, three contained coded breeders lines in their pedigrees preventing further investigation, one contained parents with unknown sensitivity (‘Emerald’. Parents: ‘Tadipor’ × ‘Macao’), one had insensitive parents (‘Cyber’. Parents: ‘Lynx’ × (‘Talon’ × ‘Beaver’), one was the sensitive landrace ‘Thatcher’, and the last (‘Ardone’) had ‘Thatcher’ in its immediate parentage.

It is possible that the low frequency of *Tsc2*-sensitive alleles in our European germplasm may reflect low occurrence of *Ptr* isolates carrying the *ToxB* gene. Indeed, *ToxB* was not present in any of 42 UK *Ptr* isolates we have recently surveyed (J. Cockram and J. Turner unpublished), indicating that sensitivity to this effector is not currently an issue in UK agricultural environments. Surveys of *Ptr* isolates from several countries have shown that presence or absence of the *ToxB* gene varies by region. For example, *ToxB* has been shown to be absent in *Ptr* from Australia (from a screen of 119 isolates; Antoni et al. 2010), New Zealand (12 isolates; Weith 2015) and Latvia, Lithuania and Romania (223 isolates; Abdullah et al. 2017), but present in Canada (Lamari et al. 2005a), Algeria (Benslimane 2018; Lamari et al. 1995; 2003), Azerbaijan, Turkey (Lamari et al. 2003), Syria, Turkey (Lamari et al. 2003; 2005b) and the USA (Abdullah 2017; Ali et al. 1999). Given that modern wheat breeding programmes often incorporate germplasm from around the world, knowledge of varietal differences in ToxB sensitivity will inform wheat breeding in regions where *Ptr* isolates carry the *ToxB* gene, or in regions predicted to be prone to incursions of *ToxB* carrying *Ptr* isolates.

Although neither of the two tetraploid wheat accessions screened here were sensitive to ToxB, the *Tsc2*-ToxB interaction has previously been reported in *T. durum*. This was first shown in the International Triticeae Mapping Initiative (ITMI) hexaploid wheat bi-parental mapping population, in which ToxB sensitivity is conferred by the synthetic hexaploid wheat line ‘W7984’ (Friesen and Faris 2004). As ‘W7984’ was developed from a cross between *T. durum* variety ‘Altar 84’ (the AB sub-genome donor) and *Aegilops tauschii* accession ‘CI 18’ (D sub-genome donor), the ToxB sensitivity on chromosome 2BS identified in the ITMI population must have originated from *T. durum*. More recently, QTL analysis of ToxB sensitivity and *Ptr* race 5 isolates in an ‘Altar’ × ‘Langdon’ durum population has demonstrated the role of ToxB sensitivity conferred by the *Tsc2* locus with tan spot susceptibility in *T. durum* (Virdi et al. 2016).

### Genetic control of ToxB sensitivity in wheat.

QTL mapping using the MAGIC and AM panels found ToxB sensitivity to be predominantly controlled by *Tsc2*. The MAGIC proximal marker originated from a wheat gene model that also contained GWAS hits 1–5 and 10. However, analysis of haplotypes around the *Tsc2* region in the AM panel unambiguously positioned *Tsc2* distal to gene model TraesCS2B01G046400 (based on SNPs Kukri_c148_1512, Kukri_c148_1346, representing GWAS hits 7 and 8, respectively) based on recombination events in ‘Orbit’ and ‘Cadogan’. The MAGIC marker immediately distal to the *Tsc2* QTL peak corresponded to AM hit 12 (Kukri_c63748_1453), with analysis of haplotypes in the AM panel positioning this marker distal to *Tsc2,* based on recombination events in four varieties: ‘Selkirk’, ‘Elka’, ‘Thatcher’ and ‘Aronde’ (Supplementary Tables 2 and 4). Therefore, the genomic regions defined by GWAS in the AM panel and genetic analyses in the MAGIC population combined to localise *Tsc2* to a 1921-kb region containing 104 gene models, based on the wheat cv. ‘Chinese Spring’ reference genome.

Within the *Tsc2* region, SNP BS00072620_51 was found to be the most significant marker in both of the genetic mapping populations investigated. Furthermore, it was shown in the AM panel (*n* = 480) to be diagnostic for ToxB sensitivity, with A:A and G:G alleles perfectly predicting insensitivity (ToxB score < 1) or sensitivity (score ≥ 1), respectively. Similarly, SNP BS00072620_51 was also diagnostic for ToxB sensitivity in the 47 varieties investigated that post-dated the AM panel. As ToxB sensitivity has been shown to be correlated with susceptibility to tan spot (Friesen and Faris 2004; Abeysekara et al. 2010; Singh et al. 2010), the co-dominant KASP marker developed here which is diagnostic for allelic state at *Tsc2* represents a useful tool for selecting against ToxB sensitivity in wheat breeding programmes in regions where ToxB-containing *Ptr* is prevalent. The incursion of isolates carrying ToxB into new areas would make marker-assisted selection against ToxB sensitivity a priority target for guarding against future changes in pathogen prevalence and virulence.

The putative minor QTL identified in the MAGIC population on the long arm of chromosome 2A differs from previously identified QTL controlling sensitivity to *Ptr* race 5 isolates located on chromosomes 2BS (*Tsr6*, Singh et al. 2010), 2AS and 2BL (Friesen and Faris 2004). Due to the low percentage of the phenotypic variation accounted for, and its identification only in the CIM–cov10 analyses, the status of this QTL is currently putative. While further investigation of this minor QTL would initially only have been thought to be of interest in determining the genetic pathways controlling the fine control of wheat ToxB sensitivity, it is interesting to note that this QTL co-locates with a robust QTL in the same MAGIC population conferring both adult plant and seedling resistance to the related necrotrophic pathogen *P. nodorum* (*QSnb.niab-2A.3* at ~ 140 cM) (Lin et al. 2019). Similarly, a minor QTL controlling sensitivity to the *P. nodorum* effector Tox3 (Downie et al. 2018) is reported to collocate with another chromosome 2A *P. nodorum* field resistance QTL*, Qsnb.niab.2A.4* (Lin et al. 2019). Collectively, these results indicate that minor necrotrophic fungal pathogen effector sensitivity QTL may also be relevant to field resistance to this class of wheat necrotrophic fungal pathogens, as has been shown for the major *P. nodorum* effector sensitivity loci *Tsn1, Snn1* and *Snn3-B1* in wheat (e.g. Friesen et al. 2009; Phan et al. 2016; Ruud et al. 2017).

### Disease resistance breeding and selection against effector sensitivity loci

The eight *Ptr* races identified are differentiated by their expression of one or a combination of the three known effectors (Lamari et al. 2003). Thus, in the context of effector sensitivity, *Ptr*–wheat interactions will depend on the allelic states at the respective loci in both the host and pathogen. It should be noted that it is likely that additional effectors remain to be discovered, and that other factors also contribute to host resistance to *Ptr* infection. Furthermore, work in another necrotrophic fungal pathogen of wheat, *P. nodorum*, has shown that the expression of one effector can suppress that of another (Phan et al. 2016). Therefore, a more complex genetic control may result from an otherwise seemingly simple underlying system. For example, while we find Chinese Spring to be insensitive to ToxB, this variety does show varying resistance/susceptibility to different *Ptr* isolates. Indeed, Tadesse et al (2006) used a *Ptr* isolate found to be virulent on Chinese Spring to screen germplasm generated by crossing 100 synthetic hexaploid wheat accessions to Chinese Spring nullisomic lines, identifying several resistance loci on chromosome 3D. Ultimately, the best strategies for increasing resistance to the widest diversity of *Ptr* strains will likely involve combined approaches, in which removal of effector sensitivity loci is complemented by genetic mapping of tan spot resistance QTL and use of linked markers for marker-assisted selection of favourable alleles.

### Future prospects towards cloning Tsc2

The current consensus is that ToxB sensitivity is predominantly conferred by a single locus, *Tsc2* (Friesen and Faris 2004; Abeysekara et al. 2010). The wheat reference genome has been constructed in the variety ‘Chinese Spring’, which is insensitive to ToxB. The only other *Ptr* effector sensitivity locus that has been cloned is *Tsn1*, at which recessive alleles confer insensitivity to the effector ToxA. The protein kinase/NBS-LRR gene underlying *Tsn1* is deleted in almost all ToxA-insensitive varieties (Faris et al. 2010), supporting the hypothesis that the gene underlying *Tsc2* may also be absent in ToxB-insensitive varieties, including ‘Chinese Spring’. To date, a small number of hexaploid wheat varieties have been resequenced, including ‘Claire’, ‘Paragon’, ‘Robigus’ (available at https://wheatis.tgac.ac.uk/grassroots-portal/blast). However, we found none of these varieties to be sensitive to ToxB. Here we investigated the genome sequence of the synthetic hexaploid wheat accession ‘W7984’ (Chapman et al. 2015), previously shown to carry a ToxB-sensitive allele at the *Tsc2* locus (Friesen and Faris 2004). While the ‘W7984’ contigs have low (55%) genome coverage and have short length (contig N50 6.7 kb), we nevertheless thought it useful to undertake an analysis of the potential gene space in a ToxB-sensitive line. This analysis allowed the identification of additional candidate genes within the ToxB-sensitive line ‘W7984’ super-contig that were not present in ToxB-insensitive variety ‘Chinese Spring’, providing potential leads for future analysis. We did not further our analysis of the ‘W7984’ genome sequence due to its fragmented nature, and because a number of projects are currently resequencing additional hexaploid bread wheat varieties. These include the 10 + Wheat Genomes Project (https://www.10wheatgenomes.com/), as well as an ongoing project to resequence all eight parents of the ‘NIAB Elite MAGIC’ population (https://gtr.ukri.org/projects?ref=BB%2FP010741%2F1). As MAGIC founder ‘Xi19’ is ToxB sensitive, we hope that the future completion of the ‘Xi19’ genome assembly will aid identification of the genetic variant(s) underlying *Tsc2*.

## Electronic supplementary material

Below is the link to the electronic supplementary material.

Supplementary Figure 1. Boxplot of mean ToxB sensitivities for the 480 varieties of the association mapping panel, according to Tsc2 haplotype groups 1 (insensitive) and 2 (sensitive). Haplotype groups are as defined in Supplementary Table 4. (TIF 3 kb)

Supplementary Figure 2. A high-resolution version of Figure 4 in which the variety names are included directly in the image. (TIF 3260 kb)

Supplementary file3 (XLSX 1113 kb)

Supplementary file4 (XLSX 54 kb)

Supplementary file5 (DOCX 20 kb)

Supplementary file6 (DOCX 14 kb)

Supplementary file7 (DOCX 19 kb)

Supplementary file8 (XLSX 47 kb)

Supplementary file9 (DOCX 15 kb)

Supplementary file10 (DOCX 25 kb)
