# Supplementary figures and images for: Genetic analysis of wheat sensitivity to the ToxB fungal effector from Pyrenophora tritici-repentis, the causal agent of tan spot
Source: Theor Appl Genet. 2020 Jan 8;133(3):935–50. doi: 10.1007/s00122-019-03517-8 (PMC7021774; doi:10.1007/s00122-019-03517-8)

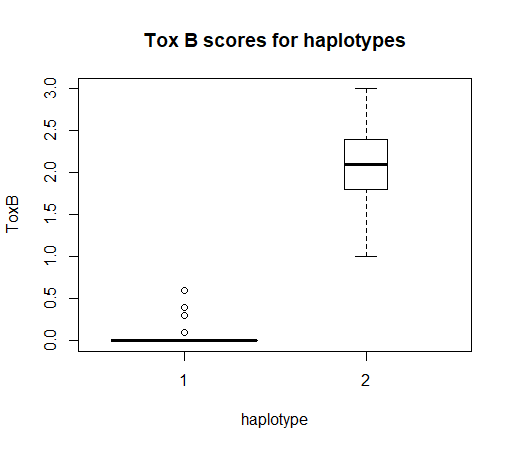

Supplement: Supplementary file 1 — Supplementary Figure 1. Boxplot of mean ToxB sensitivities for the 480 varieties of the association mapping panel, according to Tsc2 haplotype groups 1 (insensitive) and 2 (sensitive). Haplotype groups are as defined in Supplementary Table 4. (TIF 3 kb) [file 122_2019_3517_MOESM1_ESM.tif]
