# Supplementary material for: Genetic analysis of wheat sensitivity to the ToxB fungal effector from Pyrenophora tritici-repentis, the causal agent of tan spot
Source: Theor Appl Genet. 2020 Jan 8;133(3):935–50. doi: 10.1007/s00122-019-03517-8 (PMC7021774; doi:10.1007/s00122-019-03517-8)
Supplement: Supplementary file 5 — Supplementary file5 (DOCX 20 kb) [file 122_2019_3517_MOESM5_ESM.docx]

| **SNP** | **Genetic map chr^€^** | **Genetic map position^€^ (cM)** | **MAF** | ***P* value** | **-Log_10_*P*** | **IWGSC RefSeq v1.0 chr^£^** | **IWGSC RefSeq v1.0 position (bp)** | **IWGSC RefSeq v1.0 gene model** |
| --- | --- | --- | --- | --- | --- | --- | --- | --- |
| BS00070050_51 | 2B | 34.3782 | 0.079498 | 2.35E-53 | 52.62889 | 2B | 24092020 | TraesCS2B01G048500 |
| BS00075303_51 | 2B | 34.3782 | 0.079498 | 2.35E-53 | 52.62889 | 2B | 24091985 | TraesCS2B01G048500 |
| BS00072620_51 | 2B | 34.3782 | 0.079498 | 2.35E-53 | 52.62889 | 2B | 24092020 | TraesCS2B01G048500 |
| BS00072619_51a | 2B | 34.3782 | 0.080544 | 7.30E-53 | 52.1365 | 2B | 24091993 | TraesCS2B01G048500 |
| BS00070051_51 | 2B | 34.3782 | 0.082636 | 6.86E-51 | 50.16377 | 2B | 24091993 | TraesCS2B01G048500 |
| GENE_1343_556 | NA | NA | 0.081590 | 1.03E-49 | 48.98905 | 2B | 24100523^‡^ | TraesCS2B01G048700 |
| Kukri_c148_1346 | 2B | 30.84527 | 0.075314 | 8.48E-46 | 45.07148 | 2B | 23107163 | TraesCS2B01G046400 |
| Kukri_c148_1512 | 2B | 28.825 | 0.075314 | 8.48E-46 | 45.07148 | 2B | 23106301 | TraesCS2B01G046400 |
| Kukri_rep_c102899_426 | 2B | 28.825 | 0.076360 | 1.55E-45 | 44.8108 | 2B | 22784418 | TraesCS2B01G045700 |
| BS00072619_51b | 2B | 34.3782 | 0.093096 | 1.07E-44 | 43.97198 | 2B | 24091993 | TraesCS2B01G045700 |
| CAP8_c5108_139 | 2B | 49.39687 | 0.075314 | 6.19E-42 | 41.20834 | 2B | 26567970 | TraesCS2B01G054400 |
| Kukri_c63748_1453 | 2B | 34.88071 | 0.079498 | 7.63E-42 | 41.11751 | 2B | 25021434 | TraesCS2B01G051000 |
| Kukri_c148_1484 | 2B | 28.825 | 0.083682 | 2.68E-39 | 38.57108 | 2B | 23106329 | TraesCS2B01G045700 |
| BS00006788_51 | 2B | 48.39184 | 0.080544 | 4.52E-39 | 38.34514 | 2B | 16865490 | None |
| BS00085748_51 | 2B | 25.8099 | 0.066946 | 2.45E-37 | 36.61061 | 2B | 14046835 | TraesCS2B01G030700 |
| BS00022330_51 | 2B | 51.42235 | 0.069038 | 3.01E-37 | 36.52158 | 2B | 29032091 | None |
| BS00111421_51 | 2B | 51.42235 | 0.069038 | 3.01E-37 | 36.52158 | 2B | 29032050 | None |
| BS00049914_51 | 2B | 51.42235 | 0.069038 | 3.01E-37 | 36.52158 | 2B | 29032722 | None |
| BS00023025_51 | 2B | 51.42235 | 0.069038 | 3.01E-37 | 36.52158 | 2B | 29037441 | TraesCS2B01G060200 |
| BS00078519_51 | 2B | 51.42235 | 0.069038 | 3.01E-37 | 36.52158 | 2B | 29039132 | TraesCS2B01G060200 |
| BS00023718_51 | 2B | 51.42235 | 0.069038 | 3.01E-37 | 36.52158 | 2B | 29760056 | TraesCS2B01G062500 |
| BS00062696_51 | 2B | 51.42235 | 0.069038 | 3.01E-37 | 36.52158 | 2B | 29993214 | TraesCS2B01G062800 |
| BS00022572_51 | NA | NA | 0.069038 | 3.01E-37 | 36.52158 | 2B | 30499336^‡^ | TraesCS2B01G063900 |
| BS00065040_51 | 2B | 48.89436 | 0.081590 | 1.11E-36 | 35.95359 | 2B | 26581458 | TraesCS2B01G054700 |
| Excalibur_c2454_333 | 2B | 49.89939 | 0.081590 | 1.11E-36 | 35.95359 | 2B | 26581945 | TraesCS2B01G054700 |
| BS00065036_51 | 2B | 49.89939 | 0.088912 | 2.61E-34 | 33.58311 | 2B | 26581602 | TraesCS2B01G054700 |
| BS00009263_51 | 2B | 49.39687 | 0.089958 | 4.59E-33 | 32.3381 | 2B | 26564155 | TraesCS2B01G054400 |
| BS00033061_51 | 2B | 25.8099 | 0.067992 | 6.01E-33 | 32.22101 | 2B | 16857573 | TraesCS2B01G034600 |
| IACX9408 | 2B | 49.89939 | 0.088912 | 1.74E-32 | 31.76042 | 2B | 26581254 | TraesCS2B01G054700 |
| BS00034887_51 | 2B | 25.30739 | 0.069038 | 4.35E-32 | 31.3614 | 2B | 17390795 | TraesCS2B01G036600 |
| BS00100466_51 | 2B | 25.8099 | 0.069038 | 4.35E-32 | 31.3614 | 2B | 14795481 | TraesCS2B01G031700 |
| BS00088325_51a | 2B | 26.31242 | 0.069038 | 4.35E-32 | 31.3614 | 2B | 17388973 | TraesCS2B01G036600 |
| RFL_Contig1863_250 | 2B | 48.39184 | 0.092050 | 6.88E-32 | 31.16242 | 2B | 26563708 | TraesCS2B01G054400 |
| RAC875_c63883_76 | 2B | 28.825 | 0.078452 | 1.00E-31 | 30.99906 | 2B | 21951509^†^ | TraesCS2B01G044900 |
| Kukri_c23961_636 | NA | NA | 0.079498 | 2.56E-31 | 30.59157 | 2B | 21952892^‡^ | TraesCS2B01G044900 |
| Excalibur_c20007_555 | 2B | 49.89939 | 0.088912 | 3.12E-30 | 29.50528 | Un | 266170509 | TraesCSU01G330400 |
| BS00063107_51 | NA | NA | 0.094142 | 5.09E-29 | 28.29313 | 2B | 26987132^‡^ | TraesCS2B01G055300 |
| BS00063694_51 | NA | NA | 0.096234 | 5.86E-29 | 28.23225 | 2B | 26581598^‡^ | TraesCS2B01G054700 |
| Excalibur_c3789_1742 | 2B | 49.89939 | 0.096234 | 5.86E-29 | 28.23225 | Un | 397261542 | None |
| RAC875_rep_c106596_127 | 2B | 49.89939 | 0.095188 | 1.50E-28 | 27.82489 | 2B | 26752991 | TraesCS2B01G054900 |
| BS00011466_51 | 2B | 49.89939 | 0.098326 | 5.62E-28 | 27.25042 | 2B | 26588842^†^ | TraesCS2B01G06800LC |
| Excalibur_c42558_255 | NA | NA | 0.083682 | 7.26E-28 | 27.13902 | 2B | 21911596^‡^ | TraesCS2B01G044700 |
| BS00100939_51 | 2B | 51.42235 | 0.100418 | 7.11E-24 | 23.14823 | 2B | 29991152 | TraesCS2B01G062700 |
| wsnp_Ku_c48_103915 | 2B | 51.42235 | 0.100418 | 7.11E-24 | 23.14823 | 2B | 29032620 | None |
| Excalibur_c16144_185 | 2B | 51.42235 | 0.100418 | 7.11E-24 | 23.14823 | 2B | 29032619 | None |
| Kukri_c74521_181 | 2B | 51.42235 | 0.100418 | 7.11E-24 | 23.14823 | 2B | 29555408 | TraesCS2B01G061900 |
| wsnp_Ex_c1629_3103725 | 2B | 51.42235 | 0.104603 | 1.41E-23 | 22.8523 | 2B | 29037623 | TraesCS2B01G060200 |
| wsnp_Ex_c1629_3103807 | 2B | 51.42235 | 0.104603 | 1.41E-23 | 22.8523 | 2B | 29037801 | TraesCS2B01G060200 |
| BobWhite_c9843_117 | 2B | 47.3817 | 0.118201 | 1.67E-23 | 22.77823 | 2B | 26101612 | TraesCS2B01G053000 |
| BS00071650_51 | 2B | 51.42235 | 0.105649 | 1.75E-23 | 22.75665 | 2B | 29040090 | TraesCS2B01G060110LC |
| Excalibur_c46590_363 | 2B | 51.42235 | 0.101464 | 1.91E-23 | 22.71807 | 2B | 29993280 | TraesCS2B01G062800 |
| BS00011149_51 | 2B | 47.3817 | 0.118201 | 1.21E-22 | 21.91651 | 2B | 25594755 | TraesCS2B01G052300 |
| BS00048108_51a | 2B | 49.89939 | 0.080544 | 2.90E-17 | 16.53782 | 2B | 26856143^†^ | TraesCS2B01G055000 |
| Tdurum_contig30210_226b | 2B | 51.42235 | 0.130753 | 1.67E-15 | 14.776 | 2B | 28415943 | TraesCS2B01G058300 |
| Tdurum_contig41920_93a | 2B | 28.32249 | 0.116109 | 7.06E-15 | 14.1511 | 2B | 19375279 | TraesCS2B01G042400 |
| Excalibur_c1747_429 | 2B | 15.13194 | 0.152720 | 9.06E-15 | 14.04265 | 2B | 11462813 | TraesCS2B01G025000 |
| BS00010318_51 | 2B | 37.4035 | 0.304393 | 2.27E-13 | 12.64465 | 2B | 24505065^†^ | None |
| BS00070900_51 | 2B | 37.4035 | 0.304393 | 2.27E-13 | 12.64465 | 2B | 24510176 | TraesCS2B01G049400 |
| BobWhite_c25359_132 | 2B | 27.31745 | 0.123431 | 4.09E-13 | 12.38877 | 2B | 18196641^†^ | TraesCS2B01G040100 |
| BobWhite_c930_401 | 2B | 33.36806 | 0.123431 | 4.09E-13 | 12.38877 | 2B | 18386959 | TraesCS2B01G040600 |
| D_F5XZDLF01CFO7W_135 | 2B | 36.39337 | 0.248954 | 1.25E-12 | 11.90314 | 4D | 477123589 | TraesCS4D01G416400LC |
| RAC875_c87052_193 | 2B | 33.36806 | 0.126569 | 1.94E-12 | 11.71252 | 2B | 18176463 | TraesCS2B01G039900 |
| BS00077642_51 | 2B | 31.85541 | 0.170502 | 6.17E-12 | 11.21005 | 2B | 20373384 | None |
| Tdurum_contig10939_488 | NA | NA | 0.125523 | 1.17E-11 | 10.93004 | 2B | 18089739 | None |
| BS00045163_51 | 2B | 38.40853 | 0.294979 | 7.17E-11 | 10.1447 | 2B | 24950762 | TraesCS2B01G050800 |
| BS00061974_51 | 2B | 31.85541 | 0.164226 | 1.18E-10 | 9.926702 | 2B | 20077125 | None |
| BS00061979_51 | 2B | 31.85541 | 0.165272 | 1.99E-10 | 9.701741 | 2B | 20077015 | None |
| BS00066180_51 | 2B | 32.35793 | 0.180962 | 1.33E-08 | 7.876148 | 2B | 19441423 | TraesCS2B01G042700 |
| BS00066184_51 | 2B | 32.35793 | 0.182008 | 1.93E-08 | 7.714443 | 2B | 19441391 | TraesCS2B01G042700 |
| CAP11_c585_134a | 2B | 51.92487 | 0.207113 | 1.75E-07 | 6.756962 | 2B | 28416207 | TraesCS2B01G058300 |
| TA001874_1495 | 2B | 73.64541 | 0.245816 | 1.82E-07 | 6.739929 | 2B | 32963752^†^ | TraesCS2B01G066000 |
| Kukri_c30668_294 | NA | NA | 0.163180 | 2.74E-07 | 6.562249 | 2D | 9927467^‡^ | None |
| BS00014897_51 | NA | NA | 0.282427 | 4.08E-07 | 6.38934 | 2B | 17369774 | TraesCS2B01G035800LC |

**Supplementary Table 3.** SNPs identified by GWAS in the association mapping panel as being significantly (Bonferroni corrected *P*=0.01; -log10*P*>6.35) associated with ToxB sensitivity. ^€^Gardner et al. (2016). ^£^ IWGSC (2018).  ^†^The physical map position of the 2B homoeologue is reported here despite it being the second best BLASTn hit, as the marker is genetically mapped to 2B in the ‘NIAB Elite MAGIC’ genetic map described by Garner et al. (2016). ^‡^Previously predicted by Gardner et al. (2016) to map to chromosome 2B by treating the SNP as a trait and QTL mapping back to the ‘NIAB Elite MAGIC’ genetic map.
