# Supplementary material for: Genetic analysis of wheat sensitivity to the ToxB fungal effector from Pyrenophora tritici-repentis, the causal agent of tan spot
Source: Theor Appl Genet. 2020 Jan 8;133(3):935–50. doi: 10.1007/s00122-019-03517-8 (PMC7021774; doi:10.1007/s00122-019-03517-8)
Supplement: Supplementary file 6 — Supplementary file6 (DOCX 14 kb) [file 122_2019_3517_MOESM6_ESM.docx]

| **Haplotype** | **Mean ToxB sensitivity** | **Haplotype frequency** | **Kukri_rep_c102899_426** *TraesCS2B01G045700* | **Kukri_c148_1512^†^** *TraesCS2B01G046400* | **Kukri_c148_1346** *TraesCS2B01G046400* | **BS00072620_51** *TraesCS2B01G048500* | **BS00075303_51^†^** *TraesCS2B01G048500* | **BS00070050_51^†^** *TraesCS2B01G048500* | **BS00072619_51b** *TraesCS2B01G048500* | **BS00072619_51a** *TraesCS2B01G048500* | **BS00070051_51** *TraesCS2B01G048500* | **GENE_1343_556 ^†^** *TraesCS2B01G048700* | **Kukri_c63748_1453^†^** *TraesCS2B01G051000* | **CAP8_c5108_139^†^** *TraesCS2B01G054400* |
| --- | --- | --- | --- | --- | --- | --- | --- | --- | --- | --- | --- | --- | --- | --- |
| 1.1 | 0.01 | 429 | 0 | 2 | 0 | 0 | 2 | 0 | 0 | 0 | 0 | 0 | 0 | 2 |
| 1.2 | 0.00 | 1 | 0 | 2 | 0 | 0 | 2 | 0 | 0 | 0 | 0 | 0 | 0 | 0 |
| 1.3 | 0.02 | 6 | 0 | 2 | 0 | 0 | 2 | 0 | 0 | 0 | 0 | 0 | 2 | 0 |
| 1.4 | 0.00 | 2 | 0 | 2 | 0 | 0 | 2 | 0 | 0 | 0 | 0 | 0 | 2 | 2 |
| 1.5 | 0.00 | 3 | 0 | 2 | 0 | 0 | 2 | 0 | 0 | 0 | 0 | 2 | 0 | 2 |
| 1.6 | 0.00 | 1 | 0 | 2 | 0 | 0 | 2 | 0 | 2 | 2 | 0 | 0 | 0 | 2 |
| 2.1 | 2.09 | 31 | 2 | 0 | 2 | 2 | 0 | 2 | 2 | 2 | 2 | 2 | 2 | 0 |
| 2.2 | 1.90 | 2 | 0 | 2 | 0 | 2 | 0 | 2 | 2 | 2 | 2 | 2 | 2 | 0 |
| 2.3 | 1.78 | 4 | 2 | 0 | 2 | 2 | 0 | 2 | 2 | 2 | 2 | 2 | 0 | 2 |
| 2.4 | 2.00 | 1 | 2 | 0 | 2 | 2 | 0 | 2 | 2 | 2 | 2 | 2 | 2 | 2 |

**Supplementary Table 4.** Details of haplotypes across the *Tsc2* region. As defined in the association mapping panel using 12 markers derived from the wheat 90k SNP array. The IWGSC RefSeq v1.1 gene model IDs from which SNPs originate are indicated in italic. 0 = homozygous allele A:A. 2 = homozygous allele B:B.  ^†^The six markers used for the condensed six-SNP *Tsc2* region haplotype.
