# Supplementary material for: Genetic analysis of wheat sensitivity to the ToxB fungal effector from Pyrenophora tritici-repentis, the causal agent of tan spot
Source: Theor Appl Genet. 2020 Jan 8;133(3):935–50. doi: 10.1007/s00122-019-03517-8 (PMC7021774; doi:10.1007/s00122-019-03517-8)
Supplement: Supplementary file 7 — Supplementary file7 (DOCX 19 kb) [file 122_2019_3517_MOESM7_ESM.docx]

| **IWGSC RefSeq v1.0 gene ID^†^** | **Chr 2B position, gene start (bp)^†^** | **Strand^†^** | **Gene annotation^†^** | **SNPs** |
| --- | --- | --- | --- | --- |
| TraesCS2B01G046400 | 23106305 | - | U4/U6 small nuclear ribonucleoprotein Prp31 | Kukri_c148_1512, Kukri_c148_1346 |
| TraesCS2B01G046500 | 23114005 | + | ATP sulfurylase (Sulfate adenylyltransferase) |  |
| TraesCS2B01G046600 | 23118869 | + | Zinc finger (Ran-binding) family protein |  |
| TraesCS2B01G046700 | 23125028 | - | carbohydrate esterase, putative (DUF303) |  |
| TraesCS2B01G045600LC | 23131898 | + | Angiopoietin-2 |  |
| TraesCS2B01G045700LC | 23136879 | - | gamma-irradiation and mitomycin c induced 1 |  |
| TraesCS2B01G045800LC | 23286356 | + | Teneurin-4 |  |
| TraesCS2B01G045900LC | 23290583 | - | SAUR-like auxin-responsive protein family |  |
| TraesCS2B01G046000LC | 23307780 | + | Cyclolysin secretion/processing ATP-binding CyaB |  |
| TraesCS2B01G046100LC | 23309980 | + | RNA-directed DNA polymerase-related protein |  |
| TraesCS2B01G046200LC | 23310479 | + | Retrotransposon protein, putative, unclassified |  |
| TraesCS2B01G046300LC | 23312289 | + | SHAGGY-like kinase |  |
| TraesCS2B01G046400LC | 23363519 | + | Adhesion G protein-coupled receptor L1 |  |
| TraesCS2B01G046500LC | 23365365 | + | Calreticulin/calnexin |  |
| TraesCS2B01G046600LC | 23377071 | - | Ankyrin repeat and SOCS box protein 6 |  |
| TraesCS2B01G046700LC | 23466236 | - | Polynucleotidyl transferase, ribonuclease H-like superfamily |  |
| TraesCS2B01G046800 | 23533977 | + | Carboxymethylenebutenolidase 1 |  |
| TraesCS2B01G046900 | 23538092 | - | Zinc finger (CCCH-type) family protein/RNA recognition motif |  |
| TraesCS2B01G047000 | 23542051 | - | NAD(P)-binding Rossmann-fold superfamily |  |
| TraesCS2B01G047100 | 23549342 | - | Nicotianamine synthase |  |
| TraesCS2B01G047200 | 23554677 | - | Hfr-2-like protein |  |
| TraesCS2B01G046800LC | 23572596 | - | Transposon protein, putative, Mutator sub-class |  |
| TraesCS2B01G046900LC | 23574055 | - | Transposon protein, putative, Mutator sub-class |  |
| TraesCS2B01G047300 | 23576111 | + | Chitinase |  |
| TraesCS2B01G047000LC | 23576800 | - | F-box/RNI-like/FBD-like domain-containing protein |  |
| TraesCS2B01G047400 | 23580771 | - | Heat shock protein 90 |  |
| TraesCS2B01G047100LC | 23581614 | + | ATP synthase gamma chain |  |
| TraesCS2B01G047500 | 23589764 | + | caspase-6 protein |  |
| TraesCS2B01G047600 | 23596456 | - | Dirigent protein |  |
| TraesCS2B01G047700 | 23597470 | - | Dirigent protein |  |
| TraesCS2B01G047200LC | 23636228 | - | Eukaryotic translation initiation factor 3 subunit A |  |
| TraesCS2B01G047800 | 23649078 | + | Receptor-kinase, putative |  |
| TraesCS2B01G047300LC | 23668460 | + | DNA helicase |  |
| TraesCS2B01G047400LC | 23669905 | + | Transposon protein, putative, Mutator sub-class |  |
| TraesCS2B01G047500LC | 23674641 | - | Transposon protein, putative, Mutator sub-class |  |
| TraesCS2B01G047600LC | 23690832 | + | plasminogen activator inhibitor |  |
| TraesCS2B01G047700LC | 23693267 | - | 12-oxophytodienoate reductase 1 |  |
| TraesCS2B01G047900 | 23715625 | - | Glyoxylate reductase/hydroxypyruvate reductase |  |
| TraesCS2B01G047800LC | 23732229 | + | Peptide deformylase 2 |  |
| TraesCS2B01G048000 | 23736105 | - | Glyoxylate reductase/hydroxypyruvate reductase |  |
| TraesCS2B01G048100 | 23808681 | + | Glyoxylate reductase/hydroxypyruvate reductase |  |
| TraesCS2B01G047900LC | 23814840 | - | Retrotransposon protein, putative, unclassified |  |
| TraesCS2B01G048000LC | 23827241 | - | Endonuclease/exonuclease/phosphatase family |  |
| TraesCS2B01G048200 | 23878105 | + | Glyceraldehyde-3-phosphate dehydrogenase |  |
| TraesCS2B01G048100LC | 23879398 | + | S-adenosylmethionine synthase |  |
| TraesCS2B01G048200LC | 23890969 | - | B3 domain-containing protein |  |
| TraesCS2B01G048300LC | 23893866 | - | TTF-type zinc finger protein with HAT dimerization domain |  |
| TraesCS2B01G048300 | 23965542 | + | LOW protein: M-phase inducer phosphatase-like |  |
| TraesCS2B01G048400LC | 23965916 | - | Protein translocase subunit SecA |  |
| TraesCS2B01G048500LC | 23976639 | - | WD40/YVTN repeat and Bromo-WDR9-I-like domain-containing |  |
| TraesCS2B01G048600LC | 23981215 | - | Ribonuclease H-like superfamily protein |  |
| TraesCS2B01G048700LC | 23992281 | - | Transposase |  |
| TraesCS2B01G048800LC | 24003932 | + | NAD(P)H-quinone oxidoreductase chain 4, chloroplastic |  |
| TraesCS2B01G048900LC | 24032100 | - | Adenylosuccinate synthetase |  |
| TraesCS2B01G049000LC | 24040724 | - | NAD(P)H-quinone oxidoreductase chain 4, chloroplastic |  |
| TraesCS2B01G049100LC | 24050418 | + | maternal effect embryo arrest 18 |  |
| TraesCS2B01G049200LC | 24051643 | - | POU domain, class 4, transcription factor 3 |  |
| TraesCS2B01G048400 | 24089537 | + | Chalcone synthase |  |
| TraesCS2B01G048500 | 24092073 | - | Glyoxylate reductase/hydroxypyruvate reductase | **BS00070050_51**, BS00075303_51, BS00072620_51, BS00072619_51a, BS00070051_51, BS00072619_51b |
| TraesCS2B01G048600 | 24097315 | - | HXXXD-type acyl-transferase family protein |  |
| TraesCS2B01G048700 | 24099685 | - | Arginase | GENE-1343_556 |
| TraesCS2B01G048800 | 24109347 | - | HXXXD-type acyl-transferase family protein |  |
| TraesCS2B01G048900 | 24124414 | + | HXXXD-type acyl-transferase family protein |  |
| TraesCS2B01G049000 | 24134837 | + | Plant invertase/pectin methylesterase inhibitor superfamily protein, putative |  |
| TraesCS2B01G049300LC | 24141459 | + | Retrovirus-related Pol polyprotein from transposon TNT 1-94 |  |
| TraesCS2B01G049100 | 24217034 | + | HXXXD-type acyl-transferase family protein |  |
| TraesCS2B01G049200 | 24238205 | + | 3-ketoacyl-CoA thiolase |  |
| TraesCS2B01G049400LC | 24254430 | + | glyceraldehyde 3-phosphate dehydrogenaseA subu 2 |  |
| TraesCS2B01G049500LC | 24255592 | + | RNA-directed DNA polymerase |  |
| TraesCS2B01G049300 | 24264497 | - | Terpene cyclase/mutase family member |  |
| TraesCS2B01G049600LC | 24275801 | + | zinc finger MYM-type-like protein |  |
| TraesCS2B01G049700LC | 24372089 | - | Epstein-Barr virus EBNA-1-like protein |  |
| TraesCS2B01G049800LC | 24466800 | + | Potassium voltage-gated channel subfam H member 8 |  |
| TraesCS2B01G049400 | 24506582 | - | Cytochrome P450 |  |
| TraesCS2B01G049900LC | 24514916 | - | Protein-methionine-sulfoxide reductase heme-binding subunit MsrQ |  |
| TraesCS2B01G050000LC | 24515201 | - | Retrotransposon protein, putative, unclassified |  |
| TraesCS2B01G050100LC | 24518240 | - | Endonuclease/exonuclease/phosphatase family protein |  |
| TraesCS2B01G050200LC | 24519819 | - | Gag polyprotein |  |
| TraesCS2B01G049500 | 24657929 | + | Ubiquitin-like-specific protease ESD4 |  |
| TraesCS2B01G050300LC | 24669523 | - | Protein FAR1-RELATED SEQUENCE 5 |  |
| TraesCS2B01G049600 | 24673971 | + | Adenylate kinase |  |
| TraesCS2B01G049700 | 24684701 | + | UNC93-like protein 2 |  |
| TraesCS2B01G049800 | 24704724 | + | Cytochrome P450-like protein |  |
| TraesCS2B01G050400LC | 24750020 | + | Retrotransposon protein, putative, unclassified |  |
| TraesCS2B01G050500LC | 24773495 | + | Transposon protein, putative, Mutator sub-class, expressed |  |
| TraesCS2B01G050600LC | 24775489 | - | Serine/threonine protein phosphatase 7 long form isogeny |  |
| TraesCS2B01G049900 | 24817600 | + | Cytochrome P450-like protein |  |
| TraesCS2B01G050700LC | 24830381 | + | BED zinc finger,hAT family dimerization domain |  |
| TraesCS2B01G050800LC | 24831216 | + | BED zinc finger,hAT family dimerization domain |  |
| TraesCS2B01G050000 | 24852525 | + | Dirigent protein |  |
| TraesCS2B01G050100 | 24860418 | + | Dirigent protein |  |
| TraesCS2B01G050200 | 24868614 | + | Dirigent protein |  |
| TraesCS2B01G050900LC | 24905629 | + | Serpin-like protein |  |
| TraesCS2B01G050300 | 24909214 | + | Stress responsive A/B barrel domain protein |  |
| TraesCS2B01G050400 | 24912678 | + | Stress responsive A/B barrel domain protein |  |
| TraesCS2B01G050500 | 24914978 | - | Disease resistance protein (TIR-NBS-LRR class) |  |
| TraesCS2B01G050600 | 24921342 | - | dimethylallyl, adenosine tRNA methylthiotransferase |  |
| TraesCS2B01G051000LC | 24927086 | - | Ribonuclease H-like superfamily protein |  |
| TraesCS2B01G050700 | 24929361 | - | Subtilisin-like protease |  |
| TraesCS2B01G051100LC | 24941219 | - | RNA-directed DNA polymerase-related |  |
| TraesCS2B01G051200LC | 24942008 | - | Retrotransposon protein, putative, LINE subclass |  |
| TraesCS2B01G050800 | 24949285 | - | Subtilisin-like protease |  |
| TraesCS2B01G050900 | 24987456 | - | 2-oxoglutarate (2OG) and Fe(II)-dependent oxygenase superfamily protein, putative |  |
| TraesCS2B01G051000 | 25017966 | - | Cullin-associated NEDD8-dissociated protein 1 | Kukri_c63748_1453 |
| TraesCS2B01G051100 | 25069046 | + | 2-oxoglutarate (2OG) and Fe(II)-dependent oxygenase superfamily protein, putative |  |
| TraesCS2B01G051200 | 25071378 | + | MLO-like protein |  |
| TraesCS2B01G051300 | 25072123 | - | 3-oxo-5-alpha-steroid 4-dehydrogenase |  |
| TraesCS2B01G051400 | 25186030 | - | Actin-related protein 4A | BS00002660_51 |

**Supplementary Table 5.** Gene models within the *Tsc2* interval as identified in the association mapping (AM) and MAGIC populations. **^†^**IWGSC (2018). The interval defined by the AM panel: TraesCS2B01G046400 to TraesCS2B01G051000. The most significant SNP at the *Tsc2* locus, identified in both the AM panel and MAGIC population, is indicated in bold. For the markers listed here, the corresponding *P* and/or q values derived from MAGIC and AM analyse are found in Supplementary Tables 1 and 3, respectively.
