# Supplementary material for: Genetic analysis of wheat sensitivity to the ToxB fungal effector from Pyrenophora tritici-repentis, the causal agent of tan spot
Source: Theor Appl Genet. 2020 Jan 8;133(3):935–50. doi: 10.1007/s00122-019-03517-8 (PMC7021774; doi:10.1007/s00122-019-03517-8)
Supplement: Supplementary file 9 — Supplementary file9 (DOCX 15 kb) [file 122_2019_3517_MOESM9_ESM.docx]

| **Variety** | **Breeder/UK agent^†^** | **Parent 1** | **Parent 2** | **RL first appearance** | **ToxB phenotype^‡^** | **KASP genotype** |
| --- | --- | --- | --- | --- | --- | --- |
| Belgrade | Sej/SU | SJ-NA | Hereford | 2016/17 | 0 | A:A |
| Bennington | Lim | Alchemy | **Batallion** | 2017/18 | 2.2 | G:G |
| Britannia | Lim | Robigus | Cassius | 2015/16 | 0 | A:A |
| Chilton | DSV | Solstice | Option | 2013/14 | 0 | A:A |
| Costello | KWS/Sen | Timaru | **Cordiale** x **Biscay** | 2015/16 | 0 | A:A |
| Cougar | RAGT | Robigus | Tuscan | 2013/14 | 0 | A:A |
| Crusoe | Lim | **Cordialle** | Gulliver | 2012/13 | 0 | A:A |
| Cubanita | SCP/Syn | **Cordiale** | Ketchum | 2014/15 | 2.2 | G:G |
| Dickens | Secobra/Agri | Equinox x Charger | Defender | 2013/14 | 0 | A:A |
| Dunston | Els | Alchemy x Hereford | Shepherd | 2017/18 | 0 | A:A |
| Evolution | Sej/Lim | Smuggler | Robigus x SJ5558 | 2014/15 | 0 | A:A |
| Freiston | Els | Alchemy x Hereford | Shepherd | 2017/18 | 0 | A:A |
| Graham | SyP/Syn | Premio | Expert | 2016/17 | 0 | A:A |
| Hardwicke | KWS | JB Diego | Cassius | 2017/18 | 0 | A:A |
| Horatio | Lim | Alchemy | Oakley | 2012/13 | 0 | A:A |
| Icon | RAGT | Alchemy | Robigus | 2014/15 | 0 | A:A |
| KWS Barrel | KWS | Bantham | Viscount | 2016/17 | 0 | A:A |
| KWS Basset | KWS | Cassius | Scout | 2016/17 | 0 | A:A |
| KWS Crispin | KWS | Conqueror | CPBT W134 | 2016/17 | 0 | A:A |
| KWS Croft | KWS | Deben x Robigus | Robigus | 2013/14 | 0 | A:A |
| KWS Gator | KWS | Oakley | CPBT W89 | 2012/13 | 0 | A:A |
| KWS Kielder | KWS | Brompton | Oakley | 2013/14 | 0 | A:A |
| KWS Lili | KWS | **KWS Horizon** | CPBT W134 | 2015/16 | 2.2 | G:G |
| KWS Silverstone | KWS | **KWS Sterling** | JB Diego | 2016/17 | 2.5 | G:G |
| KWS Siskin | KWS | **KWS Sterling** | CPBT W134 | 2016/17 | 2.2 | G:G |
| KWS Trinity | KWS | **Grafton** | Einstein | 2015/16 | 0 | A:A |
| KWS Zyatt | KWS | **Quartz** | Hereward | 2017/18 | 2.2 | G:G |
| Leeds | Momont/KWS | Istabraq | Robigus | 2013/14 | 0 | A:A |
| LG Motown | Lim | Bantam x Huntress | Denman | 2017/18 | 0 | A:A |
| LG Sundance | Lim | Hereford x Viscount | Cassius | 2017/18 | 0 | A:A |
| Monterey | Black Ag/Sen | Istabraq | Robigus | 2013/14 | 0 | A:A |
| Moulton | Els | **Duxford** x Hereford | Shepherd | 2017/18 | 2.2 | G:G |
| Myriad | Lim | NSL00-0742 | Nijinsky | 2013/14 | 0 | A:A |
| Panacea | Lim | Robigus x Claire | Lear | 2014/15 | 0 | A:A |
| Reflection | SyP/Syn | Denman | Oakley | 2015/16 | 0 | A:A |
| Relay | RAGT | Gladiator | Victor | 2012/13 | 0 | A:A |
| RGT Conversion | RAGT | Oakley x Ambrosia | Alchemy | 2015/16 | 0 | A:A |
| RGT Gravity | RAGT | Scout | Oakley x Santiago | 2017/18 | 0 | A:A |
| RGT Illustrious | RAGT | **Batallion** | Qplus | 2016/17 | 2.2 | G:G |
| Savello | Syn | Cassius | Hereford | 2017/18 | 0 | A:A |
| Shabras | Syn | Cassius | Hereford | 2017/18 | 0 | A:A |
| Skyfall | RAGT | C4148 | Hurricane | 2014/15 | 0 | A:A |
| Spyder | Sen | Delphi | Timber | 2016/17 | 0 | A:A |
| Torch | RAGT | Moulin | Boxer | 2012/13 | 0 | A:A |
| Twister | ?/KWS | Istabraq | Robigus | 2014/15 | 0 | A:A |
| Viscount | KWS | Robigus | Canterbury | 2009/10 | 0 | A:A |
| Zulu | Lim | CEB 99080 x Claire | Robigus | 2014/15 | 0 | A:A |

**Supplementary Table 7.** Comparison of ToxB phenotype with genotypic data derived from KASP marker BS00072620_51 for 48 wheat varieties from the UK AHDB Recommended List, postdating the association mapping panel of 480 lines, and released between 2009 and 2017. Parents for each variety are indicated; parents known to be ToxB sensitive (via cross-reference to Supplementary Table 2) are indicated in bold. **^†^**Black Ag = Blackman Agriculture, DSV = DSV United Kingdom Ltd, Els = Elsoms Seeds Ltd, KWS = KWS UK Ltd, Lim = Limagrain UK Ltd, RAGT = RAGT Seeds Ltd, Sej = Sejet, Senova = Senova Seeds, Syn = Syngenta Seeds Ltd. **^‡^**Mean of five reps.
