# Supplementary material for: Genetic analysis of wheat sensitivity to the ToxB fungal effector from Pyrenophora tritici-repentis, the causal agent of tan spot
Source: Theor Appl Genet. 2020 Jan 8;133(3):935–50. doi: 10.1007/s00122-019-03517-8 (PMC7021774; doi:10.1007/s00122-019-03517-8)
Supplement: Supplementary file 10 — Supplementary file10 (DOCX 25 kb) [file 122_2019_3517_MOESM10_ESM.docx]

**Supplementary Text 1: Further details of the methods used to annotate the *Tsc2* region of the synthetic wheat line ‘W7984’**

*Liftover of gene models*

The two repeat masked sequences were aligned using Minimap2 v2.16, using the ‘Chinese Spring’ (CS) region as reference and with the command line options: "--cs=long -c -t 20 -K 500M -Y". The resulting PAF file was converted to MAF using paftools (included in Minimap2). The maf2hal and halLiftover utilities from Cactus (Paten et al. 2011; <https://github.com/ComparativeGenomicsToolkit/cactus.git>; commit d4a0e82) were then used to convert the MAF file into a suitable HAL file for the lift over of the transcript sequences. The CS GFF3 file corresponding to region 21536610-27030113 was converted to BED12 format using Mikado (Venturini et al. 2018) before using halLiftover to transfer the CS transcriptomic coordinates into a synthetic 2B assembled fragment BED12 file. A custom script remove_dupes.py, (https://github.com/lucventurini/ei-liftover) was then used to remove duplicates. Mikado was used to convert the transferred annotation into transcriptomic coordinates (BED12 format); the same was done with the original annotation from CS. Gffread v0.11.2 (https://github.com/gpertea/gffread) was used to extract the cDNA sequences of the transferred annotation. The transfer_cds.py utility from ei-liftover (https://github.com/lucventurini/ei-liftover) was used to transfer the original coding regions (CDS) to the transferred models, when possible. Gffread was used to extract the peptides and the multi_genome_compare.py from ei-liftover was then used to obtain the comparison between the original and transferred models.

*RNA-seq data*

The reads described in the Methods were trimmed to remove adapters and quality filtered using TrimGalore (Krueger F.) using commandline options: "-q 25 --clip_R1 1 --clip_R2 1 --length 50 –paired". Whole genome alignment against the full Synthetic W7984 assembly containing the synthetic 2B assembled fragment was performed using HiSat2 v2.1.0 (Kim et al. 2015) with command line options: "-q --phred33 --dta-cufflinks --max-intronlen 50000". Reads aligning to the Synth_chr2B:21536610-27030113 fragment were extracted before realignment to the Synth_chr2B:21536610-27030113 region only using both Star v2.6.1d (Dobin et al. 2013) and HiSat2 v2.1.0. These second alignments were performed using the command-line options: STAR: "--alignIntronMin 20 --alignIntronMax 10000 --alignMatesGapMax 10000 --out SAMattributes NH HI AS nM XS NM MD"; HiSat2: "--min-intronlen=20 --max-intronlen=10000".

*Transcript assembly*

The Illumina RNA-seq alignments (from STAR and HiSat2) were assembled using two different tools, StringTie and Scallop. StringTie v.1.10 (Pertea et al. 2015) was called twice, using options "-f 0.05 -m 200 -u" and "-f 0.05 -m 200", ie with and without the correction for multi-mapping reads; Scallop v0.10.3 was invoked using the option "--library_type unstranded". Both StringTie and Scallop assemblies were filtered to remove any monoexonic transcript with an estimated expression level below 1 RPKM (for Scallop) or TPM (for StringTie) using the custom script filter_assemblies_by_quant.py (https://github.com/lucventurini/ei-annotation) with the command line options: StringTie: "-q TPM -m 1 -mu 0"; Scallop: "-q RPKM -m 1 -mu 0".

*Validation of splice junctions*

RNA-seq junctions (defining introns) were derived from the RNA-seq alignments using Portcullis v1.1.2 (Mapleson et al. 2016) and the default set of filtering parameters. The junctions called by Portcullis on the different alignments were integrated into a single set using junctools, from the Portcullis suite, using the mean score across datasets as final score.

*Protein alignment*

Three protein datasets were used to help annotate the Synthetic W7984 *Tsc2* region are as follows, by aligning them to the region using GenomeThreader:

(i) Proteins from the CS42 annotation ("self"; IWGSC, 2018), extracted using GffRead 0.11.2, with parameters "-intermediate -introncutout -gcmaxgapwidth 5000 -species rice -gff3out -gcmincoverage 95 -prhdist 4 -prseedlength 7 -paralogs"

(ii) Proteins from *Aegilops tauschii* (v.4.0; Ming-Cheng 2017) and *Triticum durum* ssp. *dicoccoides* (v 1.0; Ravni 2017), from EnsEMBL plants (Kersey, 2018), with parameters: "-intermediate -introncutout -gcmaxgapwidth 5000 -species rice -gff3out -gcmincoverage 90 -prhdist 7 -prseedlength 7 -paralogs". This set was indicated with the moniker “triticeae”.

(iii) Proteins from *Oryza sativa* (IRGSP 1.0; Sakai, 2013), *Brachypodium distachyon* (v 3.0; Fox, 2013) and *Sorghum bicolor* (NCBIv3, McCormick 2017), from EnsEMBL plants, with parameters: "-intermediate -introncutout -gcmaxgapwidth 5000 -species rice -gff3out -gcmincoverage 80 -prhdist 10 -prseedlength 7 -paralogs". This set was indicated with the moniker “other”.

*Mikado*

Mikado (Venturini et al. 2018) was used to integrate the Illumina assemblies. After merging the assemblies into a coherent starting set, open reading frames (ORFs) were called on the remaining transcripts using TransDecoder v5.5.0 (Haas et al. 2013) with parameter "-m 60". Transcripts were compared with a combined database of all protein evidences, above, using DIAMOND Blastx v0.9.24 (Buchfink et al. 2014), with parameters: " --sensitive --salltitles --max-target-seqs 10 --evalue 1e-06”. In the final stage, Mikado defined gene loci and picked the best available transcript, integrating information coming from the DIAMOND Blastx, the imputed ORFs, and the junction information from Portcullis. The “athaliana_scoring.yaml” scoring file, included in the software distribution, was used to define the best transcript.

*Categorising Mikado transcripts*

In order to rank the reliability of the RNA-seq derived gene models, Mikado transcripts were compared to the liftover gene models, using Mikado compare. The predicted peptides were also compared to the original CS proteins. Transferred models were compared to the original CS gene model structures using the compare command of Mikado. Transcripts were divided as follows:

"Gold": 53 transcripts that fulfil all the following constraints:

- - Concordance (F1 == 100) with the transferred annotation (from Mikado compare)
  - AND One of the following:
    - The transferred model has an F1 of CDS junctions equal to 100% (from multi_genome_compare)
    - The transferred model has a CDS identity greater or equal to 95% (from multi_genome_compare)
    - 95% or greater reciprocal coverage of the BLASTX and 95% or greater identity
  - AND valid start and stop codon

"Silver": 51 transcripts that fulfil the following:

- - Not in the gold set
  - AND one of the following:
    - The transferred model has a CDS Identity greater than 50% AND F1 of CDS Junctions greater than 0 (from multi_genome_compare)
    - Complete junction concordance with a model, F1 of 100% (from Mikado compare)
    - BLASTX identity greater or equal than 70% and reciprocal coverage greater than 50%
  - AND junction F1 greater than 0
  - AND valid start and stop codons

"Bronze": 113 transcripts that fulfil the following:

- - Not in the gold or silver set
  - AND BLASTX query coverage greater than 0 OR Mikado compare nucleotide F1 greater than 0
- 144 transcripts with no assigned category.

*Categorising transferred annotations*

Taking the output of the multi_genome_compare.py script, transferred models were divided as follows:

- Gold, 156 transcripts:
  - Transferred F1 of the CDS exons of 100% OR CDS identity of 100%
- Silver, 28 transcripts:
  - Not Gold
  - Transferred F1 of the CDS exons greater or equal to 50% OR CDS identity greater or equal to 50%
- Bronze, 52 transcripts:
  - Not silver or gold

*Augustus hints*1. Mikado hints

Mikado hints were prepared using the categorisation defined above (see "Categorising Mikado transcripts") and the ei-annotation script prepare_mikado_hints.py (https://github.com/lucventurini/ei-annotation). Gold models were allocated a score of 10 and source M, silver models a score of 6 and a source of F, bronze and remaining models a score of 6 and 4 respectively, with source E for both.

2. Transferred annotation hints

The transferred annotation was transformed into hints using the categorisation defined above (see "Categorising transferred annotations") and the ei-annotation script prepare_mikado_hints.py. Gold models were allocated a score of 20 and source M, silver models a score of 8 and a source of F, bronze models a score of 8 and a source F, and remaining models a score of 7 and source E.

3. Portcullis junction hints

Portcullis pass junctions were transformed into hints using the ei-annotation script filter_portcullis.py. Junctions with a score of 1 (certainty of being valid according to portcullis) were assigned a score of 6, while all remaining pass junctions had a score of 4. Both sets were assigned to source E.

4. Coverage hints

BAM files from both HISAT2 and STAR were transformed into BigWig files using the bamCoverage utility from DeepTools v3.1.3 (Ramirez et al. 2014), with parameters "--ignoreDuplicates --normalizeUsing CPM --skipNAs". The resulting BigWig files were then merged using the ei-annotation script bigWigMerge.py, with parameters "-n median". The resulting wig file was converted into hints using the Augustus script wig2hints.pl, with parameters: "--width 10 --margin 10 --minthresh 2 --src=W --strand . --type=exonpart --UCSC=coverage --pri=3 --minscore=4 --prune=0.1 --radius=4.5"

5. Protein hints

Protein GFF3 files were converted to hints using the convert_proteins_to_hints.py (https://github.com/lucventurini/ei-annotation), with source P. "Self" proteins were assigned a score of 4, "Triticeae" proteins a score of 3, and all other proteins a score of 2.

6. Repeat hints

The script prepare_repeat_hits.py (https://github.com/lucventurini/ei-annotation) was used to convert the GFF3 of the repeats into Augustus hints, with source RM and score of 1.

All hints were subsequently merged together using the GenomeTools (v1.5.9) "gff3" utility.

*Augustus gene prediction*

The repeat-masked scaffold was subdivided in sixty chunks with a minimum size of 50 kbps and a minimum overlap between chunks of 10kbps, using a script included in the ei-annotation suite (split_genome_fasta.py). Subsequently, Augustus (Stanke and Morgenstern 2005) was launched on all the scaffolds using the ei-annotation wrapper "execute_augustus.py", with the Augustus command line: "augustus --species=wheat --UTR=on --extrinsicCfgFile=<EI-ANNOTATION-CFG> --stopCodonExcludedFromCDS=false --genemodel=partial --alternatives-from-evidence=true --noInFrameStop=true --allow_hinted_splicesites=atac". The extrinsic hints configuration file can be found in the ei-annotation repository (<https://tinyurl.com/y2gpq7sy>). Augustus v.3.3.2 was used for this project. Finally, the Augustus models from the chunks were merged using the Augustus utility joingenes, with parameters "-i -a". The Augustus GTF file was converted into GFF3 using ‘Convert’ in Mikado utilities. In the process, two transcript models were stripped of their CDS as the coding sequence length was not a multiple of 3 (as expected).

**Supplementary references**

Altschul SF, Gish W, Miller W, Myers EW, Lipman DJ (1990). Basic local alignment search tool. J Mol Biol, 215: 403-410.

Buchfink B, Xie C, Huson DH (2014) Fast and sensitive protein alignment using DIAMOND. Nature Methods 12:59–60.

Dobin A, Davis CA, Schlesinger F, Drenkow J, Zaleski C, Jha S, Jha S, Batut P, Chaisson M, Gingeras TR (2013) STAR: ultrafast universal RNA-seq aligner. Bioinformatics 29:15–21.

Downie RC, Bouvet L, Furuki E, Gosman N, Gardner KA, Mackay IJ, Mantello CC, Mellers G, Phan HTT, Rose GA, Tan K-C, Oliver RP, Cockram J (2018) Assessing European wheat sensitivities to *Parastagonospora nodorum* necrotrophic effectors and fine-mapping of the *Snn3-B1* locus conferring sensitivity to the effector SnTox3. Front Plant Sci 9:881.

Fox SE, Preece J, Kimbrel JA, Marchini G, Sage A, Youens-Clark K, Cruzan MB, Jaiswal P (2013) Sequencing and de novo transcriptome assembly of *Brachypodium sylvaticum* (Poaceae). Appl Plant Sci 1:1200011.

Gardner KA, Wittern LM, Mackay IJ (2016) A highly recombined, high-density, eight founder wheat MAGIC map reveals extensive segregation distortion and genomic locations of introgression segments. Plant Biotechnol J 14:1406-1417.

Haas BJ, Papanicolaou A, Yassour M, Grabherr M, Blood PD, Bowden J, Couger MB, Eccles D, Li B, Lieber M, MacManes MD, Ott M, Orvis J, Pochet N, Strozzi F, Weeks N, Westerman R, William T, Dewey CN, Henschel R, LeDuc RD, Friedman N, Regev A (2013) *De novo* transcript sequence reconstruction from RNA-seq using the Trinity platform for reference generation and analysis. Nat Proto*,* 8:1494–1512.

Hickey G, Paten D, Earl D, Zerbino D, Haussler D (2013) HAL: a hierarchical format for storing and analyzing multiple genome alignments, Bioinformatics 29:1341–1342.

Kersey PJ (2018) Ensembl Genomes 2018: an integrated omics infrastructure for non-vertebrate species. Nucleic Acids Res 46:D802–D808.

Kim D, Langmead B, Salzberg SL (2015) HISAT: a fast spliced aligner with low memory requirements. Nat Methods 12:357–360.

Krueger F. Trim Galore! Available at http://www.bioinformatics.babraham.ac.uk/projects/

trim_galore/.

Mapleson D, Venturini L, Kaithakottil G, Swarbreck D (2018) Efficient and accurate detection of splice junctions from RNA-seq with Portcullis. Gigascience 7:giy131.

McCormick RF, Truong SK, Sreedasyam A, Jenkins J, Shu S, Sims D, Kennedy M, Amirebrahimi M, Weers BD, McKinley B, Mattison A, Morishige DT, Grimwood J, Schmutz J, Mullet JE (2017) The *Sorghum bicolor* reference genome: improved assembly, gene annotations, a transcriptome atlas, and signatures of genome organization. Plant J 93:338-354.

Ming-Cheng L, Gu YQ, Puiu D, Wang H, Twardziok SO, Deal KR, Huo N, Zhu T, et al. (2017) Genome sequence of the progenitor of the wheat D genome *Aegilops tauschii*. Nature 551:498–502.

The International Wheat Genome Sequencing Consortium (IWGSC) (2018) Shifting the limits in wheat research and breeding using a fully annotated reference genome. Science 361:6403.

Pertea M, Pertea GM, Antonescu CM, Chang TC, Mendell JT, Salzberg SL (2015) Stringtie EnablesImproved Reconstruction of a Transcriptome from Rna-Seq Reads. Nat Biotechnol 3:290–295.

Ramírez F, Dündar F, Diehl S, Grüning BA, Manke T (2014) deepTools: a flexible platform for exploring deep-sequencing data. Nucleic Acids Res 42:W187–W191.

Avni R, Nave M, Barad O, Baruch K, Twardziok SO, Gundlach H, Hale I, Mascher M, et al. (2017) Wild emmer genome architecture and diversity elucidate wheat evolution and domestication. Science 357:93-97.

Sakai H, Lee SS, Tanaka T, Numa H, Kim J, Kawahara Y, Wakimoto H, Yang CC, et al. (2013) Rice Annotation Project Database (RAP-DB): an integrative and interactive database for rice genomics. Plant Cell Physiol 54:e6.

Smit AFA, Hubley R, Green P. (2013-2015) RepeatMasker Open-4.0. http://www.repeatmasker.org

Paten B, Earl D, Nguyen N, Diekhans M, Zerbino D, Haussler D (2011) Cactus: Algorithms for genome multiple sequence alignment. Genome Re*,* 21:1512-1528.

Venturini L, Kaithakottil GG, Schudoma C, Swarbreck D. ei-annotation. Available at https://github.com/lucventurini/ei-annotation
